# Supplementary material for: Cost-effectiveness of internet-based vestibular rehabilitation with and without physiotherapy support for adults aged 50 and older with a chronic vestibular syndrome in general practice
Source: BMJ Open. 2020 Oct 15;10(10):e035583. doi: 10.1136/bmjopen-2019-035583 (PMC7566722; doi:10.1136/bmjopen-2019-035583)
Supplement: Supplementary data [file bmjopen-2019-035583supp001.pdf]

Supplementary Figure 1. Overview of enrolment, allocation and follow-up.

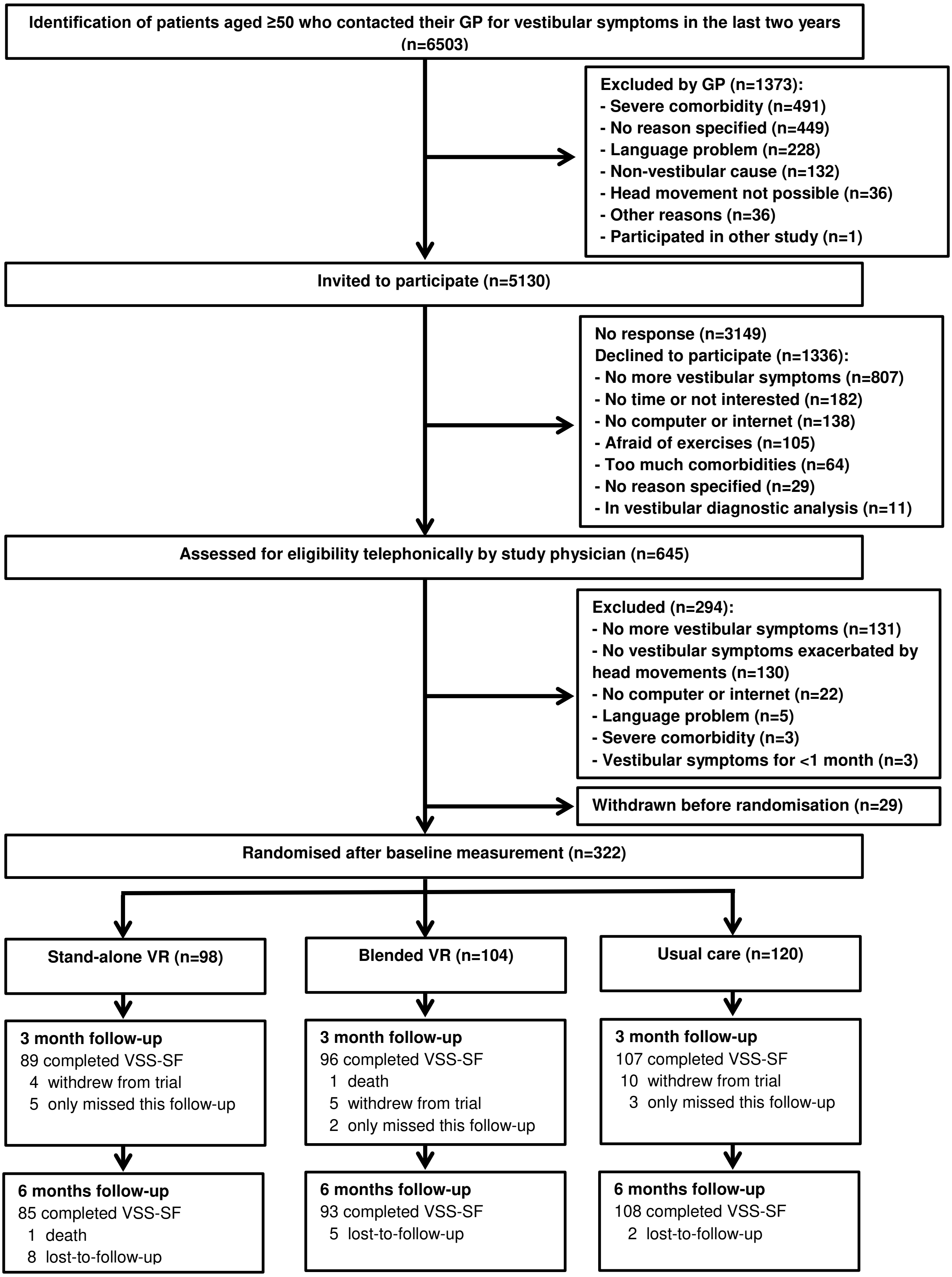

VR = vestibular rehabilitation; VSS-SF = Vertigo Symptom Scale Short Form
