# Supplementary material for: Cost-effectiveness of internet-based vestibular rehabilitation with and without physiotherapy support for adults aged 50 and older with a chronic vestibular syndrome in general practice
Source: BMJ Open. 2020 Oct 15;10(10):e035583. doi: 10.1136/bmjopen-2019-035583 (PMC7566722; doi:10.1136/bmjopen-2019-035583)
Supplement: Supplementary data [file bmjopen-2019-035583supp002.pdf]

Supplementary Table 1 Baseline characteristics. Figures are numbers (percentages) unless stated otherwise.

		Stand-alone VR (N=98)	Blended VR (N=104)	Usual care (N=120)	Total sample (N=322)
Mean (SD) age (years)		66.7 (9.5)	67.4 (9.8)	67.0 (9.4)	67.0 (9.5)
Female		64 (65)	69 (66)	64 (53)	197 (61)
Level of education					
	Low	33 (34)	37 (36)	36 (30)	106 (33)
	Middle	25 (26)	31 (30)	30 (25)	86 (27)
	High	40 (41)	36 (35)	54 (45)	130 (40)
Living situation					
	Alone	34 (35)	33 (32)	35 (29)	102 (32)
	With partner	64 (65)	71 (68)	85 (71)	220 (68)
Number of chronic diseases*					
	0	59 (60)	64 (62)	63 (53)	186 (58)
	1	28 (29)	32 (31)	41 (34)	101 (31)
	2	8 (8)	4 (4)	12 (10)	24 (7)
	≥3	3 (3)	4 (4)	4 (3)	11 (3)
Time since vestibular diagnosis**					
	One to six months	15 (15)	22 (21)	13 (11)	50 (16)
	Six months to two years	28 (29)	27 (26)	39 (33)	94 (29)
	Two years to 10 years	31 (32)	44 (42)	48 (40)	123 (38)
	More than 10 years	23 (24)	11 (11)	18 (15)	52 (16)
Self-reported vestibular diagnosis**					
	No known diagnosis	67 (68)	69 (66)	77 (64)	213 (66)
	Benign paroxysmal positional vertigo	11 (11)	17 (16)	22 (18)	50 (16)
	Meniere's disease	9 (9)	9 (9)	10 (8)	28 (9)
	Vestibular neuritis	6 (6)	4 (4)	7 (6)	17 (5)
	PPPD	0 (0)	1 (1)	0 (0)	1 (0)
	Other^	4 (4)	4 (4)	2 (2)	10 (3)
Disorders at baseline according to PHQ		14 (14)	16 (15)	23 (19)	53 (17)
	Panic disorder	2 (2)	3 (3)	5 (4)	10 (3)
	Generalised anxiety disorder	12 (12)	15 (14)	19 (16)	46 (14
	Major depressive disorder	5 (5)	6 (6)	9 (8)	20 (6)

PPPD = persistent postural-perceptual dizziness; PHQ = patient health questionnaire.
*Chronic non-specific lung disease, cardiac disease, peripheral arterial disease, stroke, diabetes mellitus, arthritis, and cancer.
**Data on this variable was missing for three participants: stand-alone VR (n=1), usual care (n=2).
^ Traumatic brain injury, cerebrovascular accident, Parkinson's disease, bacterial meningitis, and vestibular organ surgical procedures.

Supplementary Table 2 Mean costs (SE) stratified for treatment group after recoding outliers as missing (SA2) and cost outcomes (95% CI) for the intervention groups compared to usual care.

Cost category	Stand-alone VR N=98	Blended VR N=104	Usual care N=120	Difference stand-alone VR versus usual care (95% CI)	Difference blended VR versus usual care (95% CI)
Healthcare costs	846 (118)	1428 (232)	775 (103)	71 (-221 ; 368)	653 (-254 ; 1257)
Lost productivity costs	2465 (684)	2992 (766)	2083 (462)	383 (-998 ; 2199)	910 (-505 ; 3026)
Total societal costs	3311 (736)	4420 (825)	2857 (502)	454 (-1019 ; 2399)	1563 (-47 ; 3764)

SE = standard error; VR = vestibular rehabilitation; CI = confidence interval
