# Supplementary material for: Cost-effectiveness of internet-based vestibular rehabilitation with and without physiotherapy support for adults aged 50 and older with a chronic vestibular syndrome in general practice
Source: BMJ Open. 2020 Oct 15;10(10):e035583. doi: 10.1136/bmjopen-2019-035583 (PMC7566722; doi:10.1136/bmjopen-2019-035583)
Supplement: Supplementary data [file bmjopen-2019-035583supp007.pdf]

Supplementary Figure 6. SA3 – societal perspective. Cost-effectiveness acceptability curve for clinically relevant response (improvement of $\geq$ 3 points on Vertigo Symptom Scale - Short Form) comparing stand-alone vestibular rehabilitation with usual care.

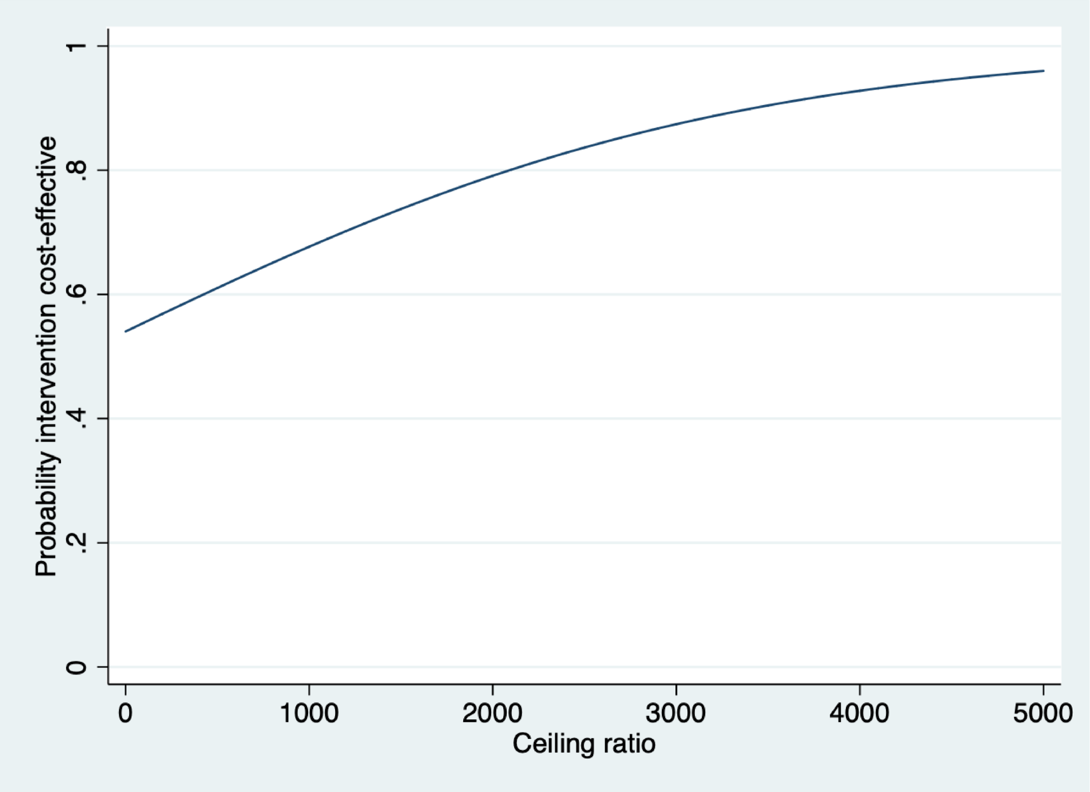

SA3 = sensitivity analysis with secondary care and medication costs excluded
