# Supplementary material for: Cost-effectiveness of internet-based vestibular rehabilitation with and without physiotherapy support for adults aged 50 and older with a chronic vestibular syndrome in general practice
Source: BMJ Open. 2020 Oct 15;10(10):e035583. doi: 10.1136/bmjopen-2019-035583 (PMC7566722; doi:10.1136/bmjopen-2019-035583)
Supplement: Author's manuscript [file bmjopen-2019-035583.draft_revisions.pdf]

BMJ Open

BMJ Open is committed to open peer review. As part of this commitment we make the peer review history of every article we publish publicly available.

When an article is published we post the peer reviewers' comments and the authors' responses online. We also post the versions of the paper that were used during peer review. These are the versions that the peer review comments apply to.

The versions of the paper that follow are the versions that were submitted during the peer review process. They are not the versions of record or the final published versions. They should not be cited or distributed as the published version of this manuscript.

BMJ Open is an open access journal and the full, final, typeset and author-corrected version of record of the manuscript is available on our site with no access controls, subscription charges or pay-per-view fees (http://bmjopen.bmj.com).

If you have any questions on BMJ Open's open peer review process please email info.bmjopen@bmj.com

BMJ Open

The cost-effectiveness of internet-based vestibular rehabilitation with and without physiotherapy support for adults aged 50 and older with a chronic vestibular syndrome in general practice

Journal:	*BMJ Open*
Manuscript ID	bmjopen-2019-035583
Article Type:	Original research
Date Submitted by the Author:	07-Nov-2019
Complete List of Authors:	van Vugt, Vincent; Amsterdam UMC - Locatie VUMC, Department of general practice and elderly care medicine; Amsterdam Public Health research institute<br>Bosmans, J; Vrije Universiteit Amsterdam, Department of Health Sciences; Amsterdam Public Health research institute<br>Finch, Aureliano; Vrije Universiteit Amsterdam, Department of Health Sciences; Amsterdam Public Health research institute<br>van der Wouden, Johannes; Amsterdam UMC - Locatie VUMC, Department of general practice and elderly care medicine; Amsterdam Public Health research institute<br>van der Horst, Henriette; Amsterdam UMC - Locatie VUMC, Department of general practice and elderly care medicine; Amsterdam Public Health research institute<br>Maarsingh, Otto; Amsterdam UMC - Locatie VUMC, Department of general practice and elderly care medicine; Amsterdam Public Health research institute
Keywords:	Health economics < HEALTH SERVICES ADMINISTRATION & MANAGEMENT, PRIMARY CARE, REHABILITATION MEDICINE, NEUROLOGY, OTOLARYNGOLOGY

SCHOLARONE™
Manuscripts

I, the Submitting Author has the right to grant and does grant on behalf of all authors of the Work (as defined in the below author licence), an exclusive licence and/or a non-exclusive licence for contributions from authors who are: i) UK Crown employees; ii) where BMJ has agreed a CC-BY licence shall apply, and/or iii) in accordance with the terms applicable for US Federal Government officers or employees acting as part of their official duties; on a worldwide, perpetual, irrevocable, royalty-free basis to BMJ Publishing Group Ltd ("BMJ") its licensees and where the relevant Journal is co-owned by BMJ to the co-owners of the Journal, to publish the Work in this journal and any other BMJ products and to exploit all rights, as set out in our licence.

The Submitting Author accepts and understands that any supply made under these terms is made by BMJ to the Submitting Author unless you are acting as an employee on behalf of your employer or a postgraduate student of an affiliated institution which is paying any applicable article publishing charge ("APC") for Open Access articles. Where the Submitting Author wishes to make the Work available on an Open Access basis (and intends to pay the relevant APC), the terms of reuse of such Open Access shall be governed by a Creative Commons licence – details of these licences and which Creative Commons licence will apply to this Work are set out in our licence referred to above.

Other than as permitted in any relevant BMJ Author's Self Archiving Policies, I confirm this Work has not been accepted for publication elsewhere, is not being considered for publication elsewhere and does not duplicate material already published. I confirm all authors consent to publication of this Work and authorise the granting of this licence.

For peer review only

The cost-effectiveness of internet-based vestibular rehabilitation with and without physiotherapy support for adults aged 50 and older with a chronic vestibular syndrome in general practice

Vincent A. van Vugt[1], Judith E. Bosmans[2], Aureliano P. Finch[2], Johannes C. van der Wouden[1], Henriëtte E. van der Horst[1], Otto R. Maarsingh[1]

[1] Amsterdam UMC, Vrije Universiteit Amsterdam, Department of general practice and elderly care medicine, Amsterdam Public Health, de Boelelaan 1117, Amsterdam, Netherlands

[2] Department of Health Sciences, Faculty of Science, Amsterdam Public Health Research Institute, Vrije Universiteit Amsterdam, Amsterdam, Netherlands.

Corresponding author:
Vincent A. van Vugt, MD
Amsterdam UMC - Location VUMC
Department of general practice and elderly care medicine
Van der Boechorststraat 7, room C-378. 1081 BT Amsterdam, The Netherlands
Phone: +31613275226
Email: v.vanvugt@amsterdamumc.nl
ORCID number: 0000-0002-2746-4820

Number of Tables: 3
Number of Figures: 2
Number of Supplementary Tables/Figures: 8
Keywords: general practice, primary care, dizziness, vertigo, vestibular rehabilitation, cost-effectiveness, economic evaluation, chronic vestibular syndrome
Pages: 22
Word count: 3865
Disclosures: none

For peer review only

ABSTRACT

Objectives To evaluate the cost-effectiveness of stand-alone and blended internet-based vestibular rehabilitation in comparison with usual care for chronic vestibular syndromes in general practice.

Design Economic evaluation alongside a three-armed, individually randomised controlled trial.

Setting 59 Dutch general practices.

Participants 322 adults, aged 50 years and older with a chronic vestibular syndrome.

Interventions Stand-alone vestibular rehabilitation consisted of a six-week, internet-based intervention with weekly online sessions and daily exercises. In blended vestibular rehabilitation, this intervention was supplemented with face-to-face physiotherapy support. Participants in the usual care group received usual general practice care without restrictions.

Main outcome measures Societal costs, quality-adjusted life years (QALY), Vertigo Symptom Scale - Short Form (VSS-SF), clinically relevant response (≥3 points VSS-SF improvement).

Results Mean societal costs in both the stand-alone and blended vestibular rehabilitation groups were statistically non-significantly higher than in the usual care group. Both intervention groups reported non-significantly more QALYs than usual care, and significantly better VSS-SF scores. For stand-alone vestibular rehabilitation compared to usual care, the probability of cost-effectiveness was 0.95 at a willingness-to-pay ratio of 24,161 €/QALY, 600 €/point improvement in VSS-SF, and 8000 €/clinically relevant responder in VSS-SF. For blended vestibular rehabilitation versus usual care, the probability of cost-effectiveness was 0.95 at a willingness-to-pay ratio of 123,335 €/QALY, 900 €/point improvement in VSS-SF and 24,000 €/clinically relevant responder in VSS-SF. In a sensitivity analysis, excluding

For peer review only - http://bmjopen.bmj.com/site/about/guidelines.xhtml

costs unlikely to be related to vestibular or internet-based VR, stand-alone VR was significantly more effective and less costly than usual care.

Conclusion Stand-alone and blended internet-based vestibular rehabilitation non-significantly increased QALYs and significantly reduced vestibular symptoms compared to usual care, while costs in both groups were non-significantly higher. Stand-alone vestibular rehabilitation has the highest probability to be cost-effective compared to usual care.

Trial registration Netherlands Trial Register NTR5712

Strengths and limitations of this study

- This is the first study to evaluate the cost-effectiveness of stand-alone internet-based vestibular rehabilitation and blended vestibular rehabilitation versus usual care for patients with a chronic vestibular syndrome in general practice.
- The economic evaluation was conducted based on the data of 322 participants aged 50 years and older with a chronic vestibular syndrome.
- Strengths of our study are the societal perspective on costs, the use of multiple outcome measures and the use of a sensitivity analysis to test robustness of our findings.
- Limitations of the study are that it was powered for the primary outcome measure VSS-SF and not for costs or QALY's, and that the follow-up was limited to six months.

For peer review only - http://bmjopen.bmj.com/site/about/guidelines.xhtml

INTRODUCTION

General practitioners frequently encounter patients with vestibular symptoms (i.e. vertigo, dizziness, vestibulovisual and postural symptoms).[1-3] The one-year prevalence of vestibular symptoms is approximately 20% in population-based studies.[1] Patients with vestibular symptoms are often unable to work, make frequent use of healthcare services and have an increased risk of falling.[1, 4, 5] The financial burden of these symptoms is therefore substantial. In a recent German study, the yearly healthcare costs were over 800 euro higher in patients with vestibular symptoms than in patients without vestibular symptoms.[5] Through assessment of timing and triggers of symptoms, patients can be classified as having either an acute vestibular syndrome, episodic vestibular syndrome or chronic vestibular syndrome.[6-8] In chronic vestibular syndromes, patients experience vestibular symptoms with features suggestive of persistent vestibular system dysfunction for months to years. The preferred treatment for chronic vestibular syndromes, according to clinical guidelines from the United States,[9, 10] the United Kingdom[11] and The Netherlands,[12] is vestibular rehabilitation (VR). VR is an exercise-based treatment developed to reduce vestibular symptoms by gradually stimulating the vestibular system.[13, 14] There is moderate to strong evidence that VR can safely and effectively reduce vestibular symptoms in both unilateral and bilateral peripheral vestibular dysfunction. [14, 15]

Despite the scientific evidence and recommendations in guidelines, VR is still underused in general practice. Surveys among Dutch [16] and English [17] general practitioners (GPs) indicate that less than 10% uses VR. Developing new ways to deliver VR in general practice may help to implement this effective treatment in daily practice. Internet interventions have several advantages over other forms of treatment: they are inexpensive, easily accessible and can be easily personalised according to the needs of individual patients. Recently, the

For peer review only - http://bmjopen.bmj.com/site/about/guidelines.xhtml

University of Southampton transformed the content of a VR booklet[18] that was shown to be (cost-)effective compared to usual care, into an internet-based VR intervention.[19] In a randomised controlled trial in the UK,[20] this stand-alone internet-based VR intervention effectively reduced vestibular symptoms compared to usual care. Combining an internet-based intervention with face-to-face support by a healthcare professional is called blended care.[21] Stand-alone internet-based interventions are generally less expensive than blended internet-based interventions, but the risk of non-adherence is also higher.[21-23] We therefore developed a blended internet-based VR intervention by adding physiotherapy support to the British internet-based VR intervention. We conducted a pragmatic, three-armed, randomised controlled trial in Dutch general practice to investigate the clinical effectiveness and cost-effectiveness of stand-alone and blended internet-based VR compared to usual care.[24] As we recently reported, both stand-alone and blended internet-based vestibular rehabilitation led to a clinically relevant and statistically significant decrease in vestibular symptoms compared to usual care.[25] The objective of the present substudy is to evaluate the cost-effectiveness of stand-alone and blended internet-based VR versus usual care for general practice patients aged 50 and older with a chronic vestibular syndrome.

For peer review only - http://bmjopen.bmj.com/site/about/guidelines.xhtml

METHODS

Design

This economic evaluation was conducted alongside a pragmatic, three-armed, randomised controlled trial in general practice. In the trial, we compared stand-alone and blended internet-based VR with usual care in patients aged 50 and older with a chronic vestibular syndrome. A detailed description of the study protocol[24] and the results of the clinical effectiveness analysis[25] can be found in previous publications. For the reporting of the cost-effectiveness analysis, we follow the recommendations in the Consolidated Health Economic Evaluation Reporting Standards (CHEERS) statement.[26]

Participants

In short, we recruited participants from 59 general practices in The Netherlands. Participants with chronic vestibular syndrome according to the ICVD were eligible for the study.[6, 7] Chronic vestibular syndrome was defined as vestibular symptoms at time of inclusion that had been present for at least one month and that were exacerbated or triggered by performing head movement. Further inclusion criteria were: age 50 years and older; good command of the Dutch language; access to the internet and an email account. Participants with an identified non-vestibular cause of dizziness, medical contraindications for making the required head movements (e.g. severe cervical arthrosis), serious comorbid conditions precluding participation in an exercise programme, or current enrolment in another study were excluded.

Interventions

Stand-alone internet-based vestibular rehabilitation (stand-alone VR)

Vertigo Training, the internet-based VR intervention we used in this trial, is a Dutch translation of the internet-based VR intervention developed by the University of

For peer review only - http://bmjopen.bmj.com/site/about/guidelines.xhtml

Southampton. The intervention lasted six weeks and consisted of six weekly online sessions with daily VR exercises. In the first session, video demonstrations and written instructions are used to teach participants the six core VR exercises. During the intervention period, participants were asked to perform these exercises for 10 minutes twice daily. Every week, the participant logged in to Vertigo Training to self-report the level of vestibular symptoms caused by each of the six exercises. Vertigo Training used this information to produce a VR exercise prescription for the coming week, tailored to the individual needs and capabilities of the participant. In addition, Vertigo Training also provides information and advice on coping and symptom control strategies which are described in more detail elsewhere.[(25)]

Blended internet-based vestibular rehabilitation with physiotherapist support (blended VR)

Supplemental to the Vertigo Training intervention, participants in the blended VR group were visited twice at home by a trained physiotherapist. These supportive physiotherapy sessions occurred in weeks one and three of the six-week intervention period and lasted for 45 minutes each.

Usual care

Participants in the usual care group received the standard level of care provided by their own GP without restrictions.

Measures

We assessed quality of life with the most widely used preference-based quality-of-life instrument in economic evaluations, [(27)] the 5-level EuroQol questionnaire (EQ-5D-5L), at baseline, and 3 and 6 months of follow-up.[(28)] We estimated utility scores by using the Dutch EQ-5D-5L tariff[(29)] and calculated Quality-Adjusted Life-Years (QALYs) by multiplying the

For peer review only - http://bmjopen.bmj.com/site/about/guidelines.xhtml

utility of a specific health state with the time spent in that health state. Transitions between health states were linearly interpolated.

We measured vestibular symptoms by the Vertigo Symptom Scale-Short Form (VSS-SF)[30, 31] at baseline, and 3 and 6 months of follow-up. The VSS-SF measures the frequency of 15 vestibular symptoms on a scale from 0 (no symptoms) to 4 (symptoms most days) during the past month (total range 0-60 points). Improvement can reflect either fewer or less frequent symptoms. In accordance with previous studies,[18, 20, 32, 33] we defined a decrease of three points or more on the VSS-SF between baseline and six month measurement as a clinically relevant response.

We measured costs from a societal perspective, using the iMTA Medical and Productivity Cost Questionnaires.[34, 35] The societal costs included healthcare costs (primary care, secondary care, medication and home care), informal care costs (i.e. costs related to help from family and friends) and lost productivity costs. Lost productivity costs consisted of costs related to absenteeism from paid and unpaid work (e.g. household activities or voluntary work), and presenteeism costs. Presenteeism is defined as reduced efficiency due to health problems while at work.[36]

We valued healthcare utilisation by using Dutch standard costs if available.[37] Otherwise, we used tariffs of professional organisations or healthcare providers themselves. We used data from the Dutch Healthcare Institute to value medication use.[38] We estimated costs of absenteeism from paid work by using the friction cost approach[39] that assumes that sick employees are replaced after a certain period of time (the friction period, i.e. 12 weeks) after which there are no productivity losses anymore. We calculated productivity losses associated

For peer review only - http://bmjopen.bmj.com/site/about/guidelines.xhtml

For peer review only

with paid work with gender-specific average wage rates of the Dutch population.[37] We assessed presenteeism by measuring participants' efficiency while at work on a numerical rating scale ranging from 0 ("I could not do anything") to 10 ("I did as much as always", i.e. the efficiency score. To calculate lost productivity hours due to presenteeism, we multiplied one minus the efficiency score with the number of days of reduced efficiency, multiplied by the number of working hours per day. We then used gender-specific average wage rates to convert the lost productivity hours due to presenteeism to lost productivity costs. To calculate lost productivity costs associated with unpaid work and informal care costs, we used a shadow price for a legally employed cleaner.[37]

Cost-effectiveness analyses

We performed the economic evaluation from a societal perspective. We compared QALYs and VSS-SF scores at 6 months pairwise between the two active treatment groups (stand-alone and blended VR) and usual care. Discounting was not necessary, because follow-up was shorter than 1 year.

We performed the analyses according to the intention to treat principle. We imputed missing cost and effect data with multiple imputation by chained equations (MICE) with predictive mean matching to account for the skewed distribution of costs.[40] The advantage of multiple imputation over single imputation methods is that the uncertainty around the imputed values is also taken into account. The predictive mean matching ensured that only observed values can be imputed. The number of imputed datasets was increased until the fraction of missing information was smaller than 5%.[41] The imputation model included variables that differed at baseline, differed between participants with and without complete follow-up, or that were associated with the outcomes (clinical effects and costs). In addition, we included all variables

For peer review only - http://bmjopen.bmj.com/site/about/guidelines.xhtml

of the analysis models in the imputation model. We performed analyses separately for each imputed dataset and then pooled applying Rubin's rules.[42]

We estimated differences in costs and effects with bivariate regression in which two separate regression models are specified, while correlation between costs and effects is maintained through correlated error terms. This allows for specification of separate covariates for costs and effects in the regression models. We used bias-corrected accelerated bootstrapping with 5,000 replications to estimate the uncertainty surrounding differences in costs and effects. We calculated incremental cost-effectiveness ratios (ICERs) by dividing the difference in costs by the difference in effects. ICERs indicate the investment that is needed to gain one unit of effect extra in the intervention group compared to control. Uncertainty surrounding the ICER is shown in cost-effectiveness (CE) planes.[43] By estimating cost-effectiveness acceptability (CEA), we subsequently combined statistical uncertainty with decision uncertainty curves to show the probability that the intervention is cost-effective compared to usual care for different willingness-to-pay ratios.[44] The willingness-to-pay ratio is defined as the maximum amount of money that society is willing to pay to gain one additional unit of effect. We employed the commonly used threshold of 20,000 € per QALY gained for appraisal of new health technologies. [45]

We conducted three sensitivity analyses to assess the robustness of the findings. In the first sensitivity analysis (SA1), we conducted the economic evaluation from the perspective of the healthcare system, meaning that only healthcare costs are included, as is recommended in some other countries, such as the UK, Belgium and Germany. In the second sensitivity analysis (SA2), we coded costs of extreme outliers (five participants who were admitted to the intensive care unit during the trial for reasons unrelated to the trial) as missing so they would

For peer review only - http://bmjopen.bmj.com/site/about/guidelines.xhtml

be imputed in the multiple imputation procedure. In the third sensitivity analysis (SA3), we excluded secondary care and medication costs. We chose to conduct SA3 because hospitalisations and expensive medications could have had a large influence on the main analysis due to relatively high costs, even though they were all judged to be unrelated to vestibular symptoms.

Patient and public involvement

Patients played an important role in the development of Vertigo Training. Detailed feedback by patients with vestibular symptoms on the content, usability and Dutch translation in prototype versions led to some amendments of the online intervention. No patients advised on interpretation of the results, nor were they involved in writing the manuscript. A lay summary of the research findings will be distributed to all participants in the study and the results will be disseminated to the relevant patient community.

For peer review only

For peer review only - http://bmjopen.bmj.com/site/about/guidelines.xhtml

RESULTS

Participants

We provide an overview of patient enrolment, allocation and follow-up in Supplementary Figure 1. Participants were recruited between June 2017 and July 2018. We randomised 322 participants at baseline: 98 participants were allocated to the stand-alone VR group, 104 to the blended VR group, and 120 to the usual care group. The baseline characteristics of participants are shown in Supplementary Table 1.

Effects

Table 1 presents the pooled mean effects and costs in the three intervention groups. For QALYs, both stand-alone and blended VR were more effective than usual care, but these differences were not statistically significant. For VSS-SF score and clinically relevant VSS-SF response, both stand-alone and blended VR were statistically significantly more effective than usual care. Overall, differences in effects for stand-alone VR versus usual care were larger than for blended VR versus usual care.

Costs

Total societal costs in both the stand-alone group (3567 €) and blended VR (3979 €) group were non-significantly higher than in the usual care group (3063 €). However, uncertainty surrounding these differences was substantial. The cost difference between stand-alone VR and usual care was smaller (i.e. mean difference 504 €, 95% CI -1082 to 2268) than between blended VR and usual care (mean difference 916 €, 95% CI -663 to 2596). Healthcare costs in both the stand-alone and blended VR groups were statistically non-significantly higher than in the usual care group. Total lost productivity costs in the stand-alone VR and usual care groups

For peer review only - http://bmjopen.bmj.com/site/about/guidelines.xhtml

were similar. Total lost productivity costs in the blended VR group were considerably – but not significantly – higher than in the usual care group.

Cost-effectiveness main analysis

Table 2 presents the results of the cost-effectiveness analyses for stand-alone VR compared with usual care. For QALYs, the ICER was 24,161 €/QALY gained for stand-alone VR in comparison with usual care. The probability of cost-effectiveness was 0.28 at a willingness-to-pay ratio of 0 €/QALY gained. At a willingness-to-pay ratio of 20,000 €/QALY gained the probability of cost-effectiveness was 0.47 for stand-alone VR versus usual care. For VSS-SF, the ICER was 132, indicating that €132 needs to be invested to gain one point of improvement in VSS-SF in the stand-alone VR group compared with usual care. The probability that stand-alone VR is cost-effective compared to usual care was 0.28 and 0.95 at willingness-to-pay ratios of 0 and 600 €/point improvement in VSS-SF, respectively (Figure 1). The ICER for response indicates that one additional participant with a clinically relevant response on the VSS-SF (≥3 points improvement) requires an investment of on average €1895 in the stand-alone VR group compared to the usual care group. At ceiling ratios of 0 and 8000 €/additional responder, the probability that stand-alone care is cost-effective in comparison with usual care was 0.28 and 0.95, respectively (Supplementary Figure 2).

In Table 3 the results of the cost-effectiveness analyses for blended VR in comparison with usual care are shown. For QALYs, the ICER was 123,335 (the large ICER is caused by the small difference in effects), meaning that €123,335 needs to be invested to gain 1 QALY in the blended VR group compared to the usual care group. The probability of cost-effectiveness was 0.14 at a ceiling ratio of 0 €/QALY gained and increased to 0.21 at a ceiling ratio of 20,000 €/QALY gained. For VSS-SF, the ICER was 280 for blended VR compared with usual

For peer review only - http://bmjopen.bmj.com/site/about/guidelines.xhtml

care, indicating that to gain one point of improvement in VSS-SF, €280 needs to be invested in blended VR compared with usual care. The CEA curve (Figure 2) shows that the probability of cost-effectiveness rapidly increases from 0.14 at a willingness-to-pay ratio of 0 €/point improvement in VSS-SF to 0.95 at a willingness-to-pay ratio of 900 €/point of improvement in VSS-SF. The ICER for response was 5599, indicating that one patient extra with a clinically relevant response on the VSS-SF (≥3 points improvement), is associated with an investment of €5599 for blended VR in comparison with usual care. The CEA curve (Supplementary Figure 3) indicates that the probability of cost-effectiveness is 0.14 and 0.95 at ceiling ratios of 0 and 24,000 €/responder extra, respectively.

Sensitivity analyses

In SA1, in which the analysis was performed from the healthcare perspective, healthcare costs in the stand-alone VR group were higher than in the usual care group, although the differences in healthcare costs were smaller than the differences in societal costs (Table 2). There was less statistical uncertainty surrounding healthcare costs compared to societal costs. In this sensitivity analysis, the ICERs for stand-alone VR compared to usual care with regard to QALYs (22,936), VSS-SF (126) and response (1799) were all slightly lower than in the main analysis (Table 2). Healthcare costs in the blended VR group were higher than in the usual care group, but smaller than the difference in societal costs in the main analysis (Table 3). As a result, in the comparison between blended VR and usual care, the ICERS for QALY's (28,848), VSS-SF (65) and response (1310) were substantially lower than in the main analysis (Table 3).

In SA2, where we coded the extreme outliers as missing and imputed their data, the societal cost difference between stand-alone VR and usual care decreased from €504 to €454

For peer review only - http://bmjopen.bmj.com/site/about/guidelines.xhtml

(Supplementary Table 2). For stand-alone VR in comparison to usual care, the ICERS for QALY's, VSS-SF and response were all markedly lower and the probability of cost-effectiveness at a willingness-to-pay ratio of 0 €/unit of effect extra increased to 0.39 in SA2 (Table 2). For blended VR compared with usual care, the societal cost difference became larger and the probabilities of cost-effectiveness lower (Table 3).

In SA3, where we excluded costs not related to vestibular symptoms, results become more positive due to smaller differences in costs between the stand-alone and blended VR groups and usual care. For blended VR in comparison with usual care, this effect is relatively small (cost difference €810 instead of €916), resulting in similar probabilities of cost-effectiveness as in the main analysis (Table 3). However, for stand-alone VR in comparison with usual care the cost difference inverts (-€75 instead of €504) (Table 2). As a result, in this analysis stand-alone VR is dominant in comparison with usual care (i.e. more effective and less expensive). CEA curves indicate that the probability of cost-effectiveness is 0.72 at a ceiling ratio of 20,000 €/QALY (Supplementary Figure 4), 0.95 at ceiling ratios of 350 €/point of improvement in VSS-SF (Supplementary Figure 5), and 0.95 at ceiling ratios of 4600 €/additional responder based on the VSS-SF (Supplementary Figure 6).

For peer review only - http://bmjopen.bmj.com/site/about/guidelines.xhtml

DISCUSSION

Principal findings

Stand-alone and blended VR resulted in a statistically non-significant gain in QALYs and significantly reduced vestibular symptoms compared to usual care. Total societal costs of both stand-alone and blended VR were statistically non-significantly higher than the costs of usual care. Since stand-alone VR was more effective and less expensive than blended VR, the probability for stand-alone VR to be cost-effective in comparison with usual care was higher. In a sensitivity analysis, where we excluded costs that were unlikely to be related to vestibular symptoms or the use of internet-based VR, stand-alone VR became dominant (i.e. more effective and less expensive) over usual care.

Comparison with existing literature

This is the first study to investigate the cost-effectiveness of internet-based VR for patients with a chronic vestibular syndrome in general practice. This internet-based VR intervention is based on the content of a VR booklet that was previously shown to be highly cost-effective compared to usual care in a randomised controlled trial.[18] Even though both internet-based and booklet-based VR interventions were inexpensive, the total costs of participants in our stand-alone VR and blended VR groups were much higher than in the study evaluating the VR booklet. This difference in costs between the trials probably occurred due to a more narrow definition of costs in the booklet VR trial. In the booklet VR trial, costs only comprised intervention costs and resource use of vestibular-related healthcare. A blinded researcher examined the medical records of participants to assess whether contacts were related to vestibular symptoms. In our trial, we analysed societal costs, which included all healthcare costs (regardless of the reason of contact), informal care costs and lost productivity costs. Total societal costs in the internet-based VR groups were considerably higher than in

For peer review only - http://bmjopen.bmj.com/site/about/guidelines.xhtml

the usual care group. The largest contributor to the difference in costs between stand-alone VR and usual care were hospital admission costs. During the trial, 7 stand-alone VR participants, 2 blended VR participants and 5 usual care participants were admitted to the hospital. Each hospitalisation was evaluated by contacting the participant and/or GP, and none of the hospitalisations were judged to be related to vestibular symptoms and/or internet-based VR. Because care in primary care is generally less expensive, this skewness in costly hospitalisations could have strongly affected the results of the cost-effectiveness analysis. Therefore, we excluded secondary care and medication costs that were likely to be unrelated to vestibular symptoms and/or internet-based VR in the third sensitivity analysis. In this analysis, stand-alone VR was dominant over usual care (i.e. more effective and less costly). For blended VR, the largest difference in costs with usual care was not seen in admissions but in loss of productivity. These relatively high absenteeism rates cannot easily be explained, but contact with a physiotherapist might have changed the way participants prioritised daily activities while experiencing vestibular symptoms. Blended VR participants may have deliberately reduced paid and unpaid work to decrease emotional distress, because they learned this can exacerbate their vestibular symptoms.(46)

Strengths and limitations

Strengths of our study are the pragmatic design of our randomised controlled trial, the societal perspective on costs, the use of multiple outcome measures (QALYs, VSS-SF, clinically relevant response) and conduct of three sensitivity analyses in addition to the main analysis to assess the robustness of the results.

There are also several limitations. First, the trial was powered for the primary outcome measure VSS-SF and not for costs or QALYs. This is reflected in a substantial uncertainty surrounding the relatively large cost differences between groups that did not reach statistical

For peer review only - http://bmjopen.bmj.com/site/about/guidelines.xhtml

significance. Secondly, the effectiveness and costs were measured over six months. Based on this study, we cannot ascertain the long-term cost-effectiveness of stand-alone and blended VR versus usual care. Nevertheless, an increase in cost-effectiveness might occur in the long term. Previous VR trials have also shown effectiveness at twelve months,[14, 18] and costs for internet-based VR are not expected to increase during a longer follow-up period. Thirdly, the differences measured in QALYs between the intervention groups and usual care were not significant, unlike the differences in VSS-SF scores. The EQ-5D-5L, a generic utility measure, may not have been sensitive enough to capture changes in quality of life in our population. Certain domains that are important for patients with chronic vestibular syndromes, such as the disease's unpredictability and its impact on social and role functioning, are not measured by the EQ-5D-5L.[47, 48] We also used the Dizziness Handicap inventory[49, 50] in our trial, an outcome measure that specifically quantifies the impact on daily life by vestibular symptoms. We did find significant differences in Dizziness Handicap Inventory scores favouring stand-alone VR and blended VR over usual care (adjusted mean difference at 6 months: -4.9 points; 95% CI -8.4 to -1.3, and -4.5 points; 95% CI -8.0 to -0.9).[25] Using the EQ-5D-5L might have caused us to underestimate the impact of stand-alone and blended VR on quality of life.

Conclusions and implications for research and/or practice

Stand-alone and blended internet-based VR both led to a statistically non-significant gain in QALYs and a significant reduction of vestibular symptoms, but the costs were (non-significantly) higher compared to usual care. After excluding costs unlikely to be related to vestibular symptoms or internet-based VR, stand-alone VR became dominant over usual care in the cost-effectiveness analysis (i.e. more effective and less costly). Internet-based VR is an easily accessible, effective form of treatment that could potentially improve care for a largely

For peer review only - http://bmjopen.bmj.com/site/about/guidelines.xhtml

For peer review only

undertreated group of patients with a chronic vestibular syndrome in general practice. Based on the results of this economic evaluation, stand-alone VR should be preferred over blended VR.

For peer review only

For peer review only - http://bmjopen.bmj.com/site/about/guidelines.xhtml

ADDITIONAL INFORMATION

Competing interests

All authors have completed the Unified Competing Interest form (available on request from the corresponding author) and declare: support from The Netherlands Organisation for Health Research and Development (ZonMw); no financial relationships with any organisations that might have an interest in the submitted work in the previous three years, no other relationships or activities that could appear to have influenced the submitted work.

Funding

This study was funded by The Netherlands Organisation for Health Research and Development (ZonMw) (programme Quality in health care; grant number 839110015). The sponsors did not participate in the study design, data collection, analysis, interpretation, or the preparation or submission of this report.

Ethical approval

The study protocol was approved by the Medical Ethics Committee of the VU University Medical Center. All participants included in the study provided written informed consent.

Copyright

The Corresponding Author has the right to grant on behalf of all authors and does grant on behalf of all authors, an exclusive licence (or non exclusive for government employees) on a worldwide basis to the BMJ Publishing Group Ltd to permit this article (if accepted) to be published in BMJ editions and any other BMJPGL products and sublicences such use and exploit all subsidiary rights, as set out in our licence.

For peer review only - http://bmjopen.bmj.com/site/about/guidelines.xhtml

Transparency declaration

Vincent van Vugt affirms that this manuscript is an honest, accurate, and transparent account of the study being reported; that no important aspects of the study have been omitted; and that any discrepancies from the study as planned (and, if relevant, registered) have been explained.

Contributors

Contributors: ORM obtained the funding and coordinated the study. ORM, VAvV, JCvdW, HEvdH and JEB were involved in the design of the study. VAvV collected the data. JEB and APF analysed the data. VAvV and JEB wrote the first substantial draft of the article and VAvV is the guarantor. ORM, JCvdW, JEB, APF and HEvdH critically revised the manuscript. All authors read and approved the final manuscript.

For peer review only

TABLES

Table 1 Pooled mean effect and cost outcomes (SE) stratified for treatment group and differences in mean effect and cost outcomes (95% CI) for the intervention groups compared to usual care.

**Outcome**	**Stand-alone VR N=98**	**Blended VR N=104**	**Usual care N=120**	**Difference stand-alone VR versus usual care**	**Difference blended VR versus usual care**
Effects					
QALY	0.43 (0.008)	0.41 (0.008)	0.41 (0.008)	0.02 (-0.001 ; 0.04)	0.01 (-0.01 ; 0.03)
VSS-SF	8.1 (0.91)	8.5 (0.70)	11.4 (0.95)	3.8 (1.7 ; 6.0)	3.3 (1.3 ; 5.2)
Response	0.72 (0.05)	0.61 (0.05)	0.45 (0.95)	0.27 (0.13 ; 0.40)	0.16 (0.02 ; 0.30)
Costs					
Intervention	39 (0)	155 (5)	0 (0)	39	155
Total healthcare	1379 (377)	1116 (229)	901 (162)	478 (-100 ; 1514)	215 (-272 ; 836)
Primary care	304 (42)	362 (59)	315 (37)	-11 (-112 ; 97)	47 (-72 ; 200)
Complementary medicine	22 (7)	8 (3)	29 (11)	-7 (-37 ; 12)	-21 (-54 ; -6)
Outpatient care	104 (19)	147 (78)	116 (20)	-12 (-62 ; 37)	31 (-62 ; 344)
Admissions	682 (350)	22 (19)	129 (95)	553 (102 ; 1563)	-107 (-471 ; 2)
Medication	209 (78)	352 (172)	171 (57)	38 (-105 ; 288)	181 (-46 ; 817)
Home care	58 (22)	224 (107)	140 (43)	-82 (-193 ; -5)	84 (-59 ; 460)
Informal care	88 (30)	187 (93)	112 (38)	-24 (-123 ; 56)	75 (-53 ; 392)
Total lost productivity	2061 (538)	2521 (541)	2049 (443)	12 (-1260 ; 1439)	472 (-846 ; 1825)
Absenteeism	241 (133)	931 (331)	486 (199)	-245 (-776 ; 190)	445 (-186 ; 1337)
Presenteeism	10 (3)	14 (5)	15 (5)	-5 (-19 ; 4)	-1 (-15 ; 12)
Unpaid work	1810 (518)	1576 (229)	1548 (361)	262 (-807 ; 1618)	28 (-981 ; 1069)
Total societal	3567 (701)	3979 (667)	3063 (520)	504 (-1082 ; 2268)	916 (-663 ; 2596)

SE = standard error; CI = confidence interval; VR = vestibular rehabilitation; VSS-SF = Vertigo Symptom Scale – Short Form, range 0-60, clinically relevant difference 3 points; Response = percentage of participants with a decrease of ≥3 points in VSS-SF between baseline and six months

For peer review only - http://bmjopen.bmj.com/site/about/guidelines.xhtml

Table 2 Cost-effectiveness outcomes for stand-alone VR compared to usual care.

Outcome	ΔC (95% CI)	ΔE (95% CI)	ICER	CE plane			
				NE	SE	SW	NW
**Main analysis – societal perspective**							
QALY	504 (-1064 ; 2303)	0.02 (-0.001 ; 0.04)	24,161	69%	28%	0%	3%
VSS-SF	504 (-1052 ; 2294)	3.8 (1.7 ; 6.0)	132	72%	28%	0%	0%
Response*	504 (-1057 ; 2282)	0.27 (0.13 ; 0.40)	1895	72%	28%	0%	0%
**SA1 – healthcare perspective**							
QALY	478 (-103 ; 1510)	0.02 (-0.001 ; 0.04)	22,936	86%	11%	0%	3%
VSS-SF	478 (-94 ; 1527)	3.8 (1.7 ; 6.0)	126	89%	11%	0%	0%
Response*	478 (-104 ; 1520)	0.27 (0.13 ; 0.40)	1799	89%	11%	0%	0%
**SA2 – societal perspective, outliers recoded as missings**							
QALY	241 (-1194 ; 1838)	0.02 (-0.001 ; 0.04)	11,388	58%	39%	0%	3%
VSS-SF	241 (-1184 ; 1851)	3.7 (1.6 ; 5.7)	66	60%	40%	0%	0%
Response*	241 (-1195 ; 1832)	0.27 (0.13 ; 0.40)	910	61%	39%	0%	0%
**SA3 – societal perspective, only costs related to vestibular symptoms**							
QALY	-75 (-1425 ; 1450)	0.02 (-0.001 ; 0.04)	-3603	43%	54%	1%	2%
VSS-SF	-75 (-1430 ; 1463)	3.8 (1.7 ; 6.0)	-20	45%	55%	0%	0%
Response*	-75 (-1432 ; 1453)	0.27 (0.13 ; 0.40)	-283	45%	55%	0%	0%

* Response was defined as ≥3 points improvement on the Vertigo Symptom Scale – Short Form after six months
ΔC = cost difference between stand-alone VR and usual care; ΔE = effect difference between stand-alone VR and usual care; CI = confidence interval; ICER = incremental cost-effectiveness ratio; CE = cost-effectiveness; NE = northeast (more expensive & more effective); SA1 = sensitivity analysis with only healthcare costs included; SA2 = sensitivity analysis with extreme outliers recoded as missings; SA3 = sensitivity analysis with secondary care and medication costs excluded; SE = southeast (less expensive & more effective); SW = southwest (less expensive & less effective); NW = northwest (more expensive & less effective); VR = vestibular rehabilitation; VSS-SF = Vertigo Symptom Scale – Short Form; QALY = quality-adjusted life-years

For peer review only - http://bmjopen.bmj.com/site/about/guidelines.xhtml

Table 3 Cost-effectiveness outcomes for blended VR compared to usual care.

Outcome	ΔC (95% CI)	ΔE (95% CI)	ICER	CE plane			
				NE	SE	SW	NW
**Main analysis – societal perspective**							
QALY	916 (-660 ; 2579)	0.007 (-0.01 ; 0.03)	123,335	62%	12%	1%	25%
VSS-SF	916 (-655 ; 2610)	3.3 (1.3 ; 5.2)	280	86%	14%	0%	0%
Response*	916 (-658 ; 2583)	0.16 (0.02 ; 0.30)	5599	85%	13%	0%	0%
**SA1 – healthcare perspective**							
QALY	215 (-273 ; 820)	0.007 (-0.01 ; 0.03)	28,848	55%	19%	3%	23%
VSS-SF	215 (-263 ; 828)	3.3 (1.4 ; 5.2)	65	78%	22%	0%	0%
Response*	215 (-263 ; 832)	0.16 (0.02 ; 0.30)	1310	77%	22%	0%	1%
**SA2 – societal perspective, outliers recoded as missings**							
QALY	1140 (-404 ; 2811)	0.007 (-0.01 ; 0.03)	156,954	66%	8%	1%	25%
VSS-SF	1140 (-404 ; 2811)	3.1 (1.3 ; 5.0)	366	91%	9%	0%	0%
Response*	1140 (-401 ; 2789)	0.16 (0.02 ; 0.30)	6988	91%	8%	0%	1%
**SA3 – societal perspective, only costs related to vestibular symptoms**							
QALY	810 (-653 ; 2400)	0.007 (-0.01 ; 0.03)	109,121	61%	13%	1%	24%
VSS-SF	810 (-653 ; 2406)	3.3 (1.4 ; 5.2)	248	85%	15%	0%	0%
Response*	810 (-650 ; 2410)	0.16 (0.02 ; 0.30)	4954	84%	15%	0%	1%

* Response was defined as ≥3 points improvement on the Vertigo Symptom Scale – Short Form after six months
ΔC = cost difference between stand-alone VR and usual care; ΔE = effect difference between stand-alone VR and usual care; CI = confidence interval; ICER = incremental cost-effectiveness ratio; CE = cost-effectiveness; NE = northeast (more expensive & more effective); SA1 = sensitivity analysis with only healthcare costs included; SA2 = sensitivity analysis with extreme outliers recoded as missings; SA3 = sensitivity analysis with secondary care and medication costs excluded; SE = southeast (less expensive & more effective); SW = southwest (less expensive & less effective); NW = northwest (more expensive & less effective); VR = vestibular rehabilitation; VSS-SF = Vertigo Symptom Scale – Short Form; QALY = quality-adjusted life-years

For peer review only - http://bmjopen.bmj.com/site/about/guidelines.xhtml

REFERENCES

1. Neuhauser HK. The epidemiology of dizziness and vertigo. Handb Clin Neurol. 2016;137:67-82.
2. Hanley K, O' Dowd T. Symptoms of vertigo in general practice: a prospective study of diagnosis. Br J Gen Pract. 2002;52(483):809-12.
3. Bisdorff A, Von Brevern M, Lempert T, et al. Classification of vestibular symptoms: towards an international classification of vestibular disorders. J Vestib Res. 2009;19(1-2):1-13.
4. Saber Tehrani AS, Coughlan D, Hsieh YH, et al. Rising annual costs of dizziness presentations to U.S. emergency departments. Acad Emerg Med. 2013;20(7):689-96.
5. Wang X, Strobl R, Holle R, et al. Vertigo and dizziness cause considerable more health care resource use and costs: results from the KORA FF4 study. J Neurol. 2019.
6. Bisdorff A. Vestibular symptoms and history taking. Handb Clin Neurol. 2016;137:83-90.
7. Bisdorff AR, Staab JP, Newman-Toker DE. Overview of the International Classification of Vestibular Disorders. Neurol Clin. 2015;33(3):541-50, vii.
8. Edlow JA, Gurley KL, Newman-Toker DE. A New Diagnostic Approach to the Adult Patient with Acute Dizziness. J Emerg Med. 2018;54(4):469-83.
9. Bhattacharyya N, Gubbels SP, Schwartz SR, et al. Clinical Practice Guideline: Benign Paroxysmal Positional Vertigo (Update). Otolaryngol Head Neck Surg. 2017;156(3_suppl):S1-S47.
10. Hall CD, Herdman SJ, Whitney SL, et al. Vestibular rehabilitation for peripheral vestibular hypofunction: an evidence-based clinical practice guideline: from the American Physical Therapy Association Neurology section. J Neurol Phys Ther. 2016;40(2):124-55.
11. National Institute for Health and Care Excellence (NICE) Vestibular neuronitis. Nice, 2011. [https://cks.nice.org.uk/vestibular-neuronitis]
12. Summary of the Dutch College of General Practitioners' practice guideline 'Dizziness' (in Dutch). Utrecht: Dutch college of general practitioners, 2017. [https://www.nhg.org/system/files/content/nhg_org/uploads/standaard/pdf/m75_2.pdf 2017]
13. van Vugt VA, van der Horst HE, Payne RA, Maarsingh OR. Chronic vertigo: treat with exercise, not drugs. BMJ. 2017;358:j3727.
14. McDonnell MN, Hillier SL. Vestibular rehabilitation for unilateral peripheral vestibular dysfunction. Cochrane Database Syst Rev. 2015;1:CD005397.
15. Porciuncula F, Johnson CC, Glickman LB. The effect of vestibular rehabilitation on adults with bilateral vestibular hypofunction: a systematic review. J Vestib Res. 2012;22(5-6):283-98.
16. van Vugt VA, Diaz Nerio PM, van der Wouden JC, et al. Use of canalith repositioning manoeuvres and vestibular rehabilitation: a GP survey. Scand J Prim Health Care. 2017;35(1):19-26.
17. Jayarajan V, Rajenderkumar D. A survey of dizziness management in General Practice. J Laryngol Otol. 2003;117(8):599-604.
18. Yardley L, Barker F, Muller I, et al. Clinical and cost effectiveness of booklet based vestibular rehabilitation for chronic dizziness in primary care: single blind, parallel group, pragmatic, randomised controlled trial. BMJ. 2012;344:e2237.
19. Geraghty AW, Kirby S, Essery R, et al. Internet-based vestibular rehabilitation for adults aged 50 years and over: a protocol for a randomised controlled trial. BMJ Open. 2014;4(7):e005871.

For peer review only - http://bmjopen.bmj.com/site/about/guidelines.xhtml

20. Geraghty AWA, Essery R, Kirby S, et al. Internet-based vestibular rehabilitation for older Adults with chronic dizziness: a randomized controlled trial in primary care. Ann Fam Med. 2017;15(3):209-16.

21. van der Vaart R, Witting M, Riper H, et al. Blending online therapy into regular face-to-face therapy for depression: content, ratio and preconditions according to patients and therapists using a Delphi study. BMC Psychiatry. 2014;14:355.

22. van Ballegooijen W, Cuijpers P, van Straten A, et al. Adherence to Internet-based and face-to-face cognitive behavioural therapy for depression: a meta-analysis. PLoS One. 2014;9(7):e100674.

23. Wilhelmsen M, Lillevoll K, Risor MB, et al. Motivation to persist with internet-based cognitive behavioural treatment using blended care: a qualitative study. BMC Psychiatry. 2013;13:296.

24. van Vugt VA, van der Wouden JC, Bosmans JE, et al. Guided and unguided internet-based vestibular rehabilitation versus usual care for dizzy adults of 50 years and older: a protocol for a three-armed randomised trial. BMJ Open. 2017;7(1):e015479.

25. van Vugt VA, van der Wouden JC, Essery R, et al. Internet based vestibular rehabilitation with and without physiotherapy support for adults aged 50 and older with a chronic vestibular syndrome in general practice: three armed randomised controlled trial. BMJ. 2019;367:l5922.

26. Husereau D, Drummond M, Petrou S, et al. Consolidated Health Economic Evaluation Reporting Standards (CHEERS) statement. BMJ. 2013;346:f1049.

27. Richardson J. Multiattribute utility instruments and their use. Encyclopedia of Health Economics San Diego: Elsevier Science; 2014.

28. Herdman M, Gudex C, Lloyd A, et al. Development and preliminary testing of the new five-level version of EQ-5D (EQ-5D-5L). Qual Life Res. 2011;20(10):1727-36.

29. Versteegh M, Vermeulen K, Evers S, et al. Dutch tariff for the five-level version of EQ-5D. Value Health. 2016;19(4):343-52.

30. Yardley L, Masson E, Verschuur C, et al. Symptoms, anxiety and handicap in dizzy patients: development of the vertigo symptom scale. J Psychosom Res. 1992;36(8):731-41.

31. Wilhelmsen K, Strand LI, Nordahl SH, et al. Psychometric properties of the Vertigo symptom scale - Short form. BMC Ear Nose Throat Disord. 2008;8:2.

32. Yardley L, Donovan-Hall M, Smith HE, et al. Effectiveness of primary care-based vestibular rehabilitation for chronic dizziness. Ann Intern Med. 2004;141(8):598-605.

33. Yardley L, Kirby S. Evaluation of booklet-based self-management of symptoms in Meniere disease: a randomized controlled trial. Psychosom Med. 2006;68(5):762-9.

34. Bouwmans C, Hakkaart-van Roijen L, Koopmanschap M, et al. Handleiding *i*MTA Medical Cost Questionnaire (*i*MCQ). 2013.

35. Bouwmans C, Krol M, Severens H, et al. The iMTA Productivity Cost Questionnaire: A Standardized Instrument for Measuring and Valuing Health-Related Productivity Losses. Value Health. 2015;18(6):753-8.

36. Aronsson G, Gustafsson K, Dallner M. Sick but yet at work. An empirical study of sickness presenteeism. J Epidemiol Community Health. 2000;54(7):502-9.

37. Hakkaart-van Roijen L, Tan S, Bouwmans C. Handleiding voor kostenonderzoek [Dutch manual for costing in economic evaluations]. . 2011.

38. Medicijnkosten Nederland [Internet]. Medicijnkosten.nl. 2019 [cited 6 November 2019]. Available from: http://www.medicijnkosten.nl/

39. Koopmanschap MA, Rutten FF, van Ineveld BM, et al. The friction cost method for measuring indirect costs of disease. J Health Econ. 1995;14(2):171-89.

40. White IR, Royston P, Wood AM. Multiple imputation using chained equations: Issues and guidance for practice. Stat Med. 2011;30(4):377-99.

For peer review only - http://bmjopen.bmj.com/site/about/guidelines.xhtml

41. Madley-Dowd P, Hughes R, Tilling K, et al. The proportion of missing data should not be used to guide decisions on multiple imputation. J Clin Epidemiol. 2019;110:63-73.

42. Rubin D. Multiple imputation for nonresponse in surveys. Hoboken, N.J.: John Wiley; 2011

43. Black WC. The CE plane: a graphic representation of cost-effectiveness. Med Decis Making. 1990;10(3):212-4.

44. Fenwick E, O'Brien BJ, Briggs A. Cost-effectiveness acceptability curves--facts, fallacies and frequently asked questions. Health Econ. 2004;13(5):405-15.

45. Cost-effectiveness in practice: National Health Care Institute of The Netherlands; 2015 [Available from: https://english.zorginstituutnederland.nl/publications/reports/2015/06/16/cost-effectiveness-in-practice.]

46. Meli A, Zimatore G, Badaracco C, et al. Effects of vestibular rehabilitation therapy on emotional aspects in chronic vestibular patients. J Psychosom Res. 2007;63(2):185-90.

47. Ciorba A, Bianchini C, Scanelli G, et al. The impact of dizziness on quality-of-life in the elderly. Eur Arch Otorhinolaryngol. 2017;274(3):1245-50.

48. Finch AP, Brazier JE, Mukuria C, et al. An Exploratory Study on Using Principal-Component Analysis and Confirmatory Factor Analysis to Identify Bolt-On Dimensions: The EQ-5D Case Study. Value Health. 2017;20(10):1362-75.

49. Jacobson GP, Newman CW. The development of the Dizziness Handicap Inventory. Arch Otolaryngol Head Neck Surg. 1990;116(4):424-7.

50. Tamber AL, Wilhelmsen KT, Strand LI. Measurement properties of the Dizziness Handicap Inventory by cross-sectional and longitudinal designs. Health Qual Life Outcomes. 2009;7:101.

For peer review only

For peer review only - http://bmjopen.bmj.com/site/about/guidelines.xhtml

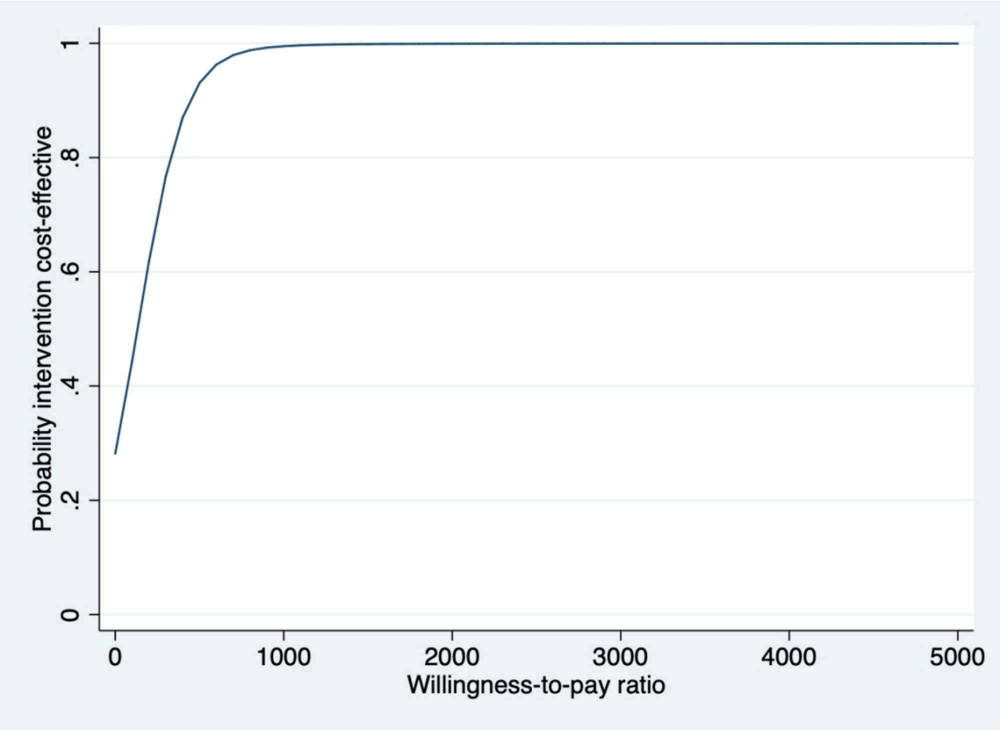

Figure 1. Main analysis – societal perspective. Cost-effectiveness acceptability curve for the Vertigo Symptom Scale - Short Form comparing stand-alone VR with usual care.
VR = vestibular rehabilitation

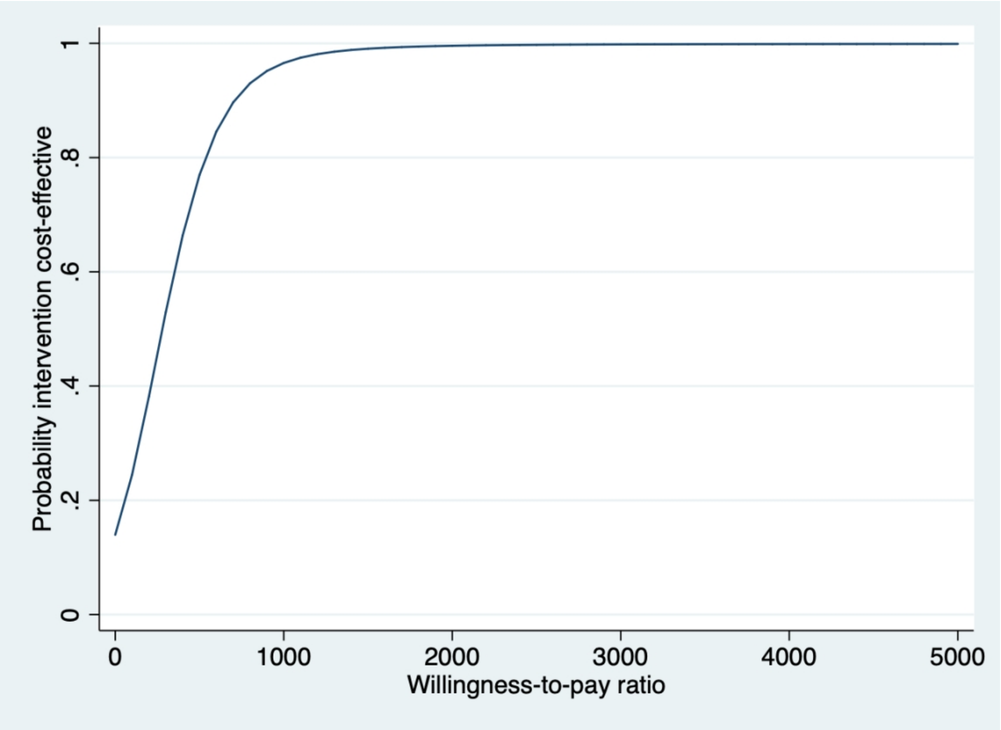

Figure 2. Main analysis – societal perspective. Cost-effectiveness acceptability curve for the Vertigo Symptom Scale - Short Form comparing blended VR with usual care.
VR = vestibular rehabilitation

Supplementary Figure 1. Overview of enrolment, allocation and follow-up.

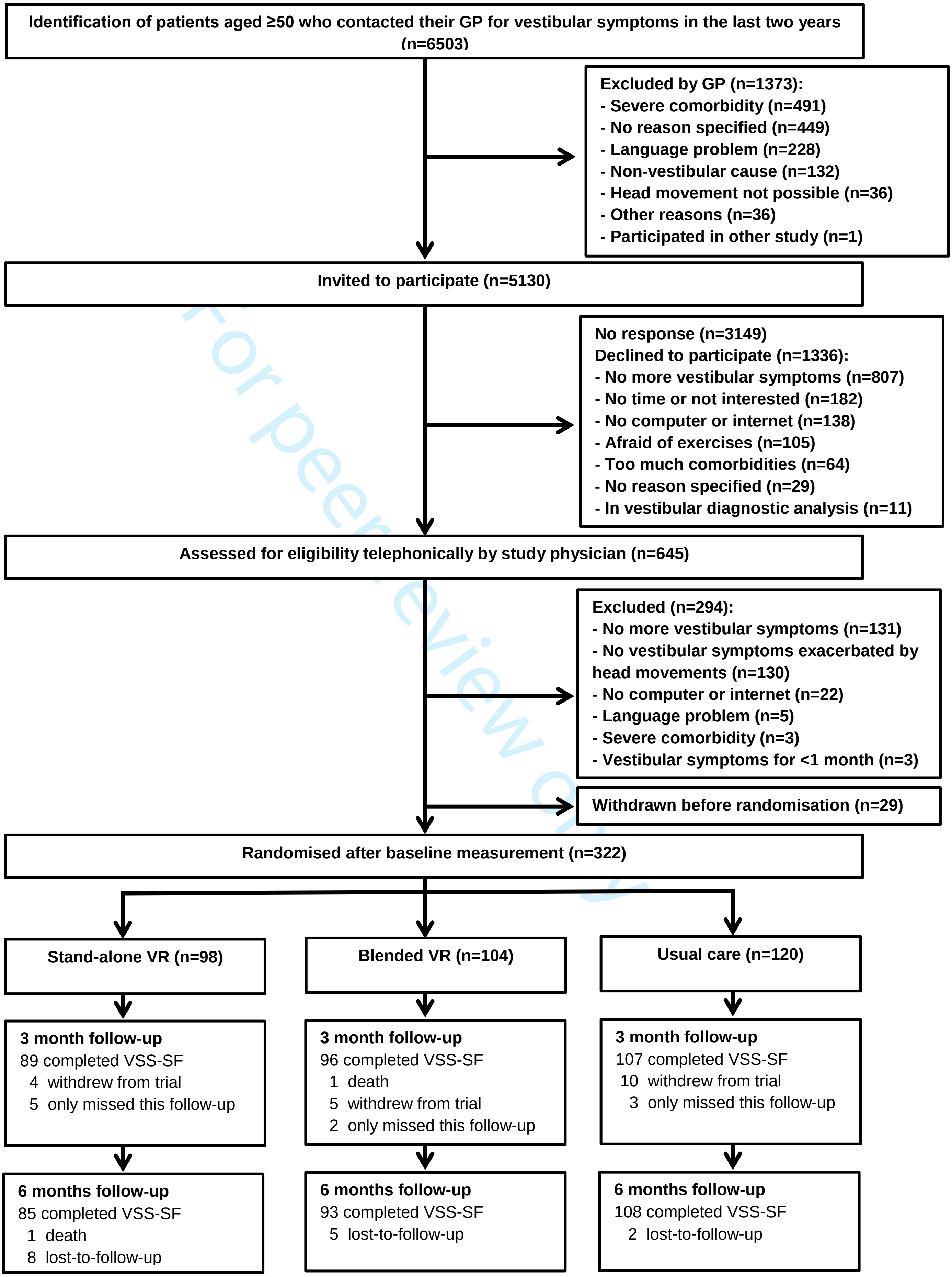

VR = vestibular rehabilitation; VSS-SF = Vertigo Symptom Scale Short Form

For peer review only - http://bmjopen.bmj.com/site/about/guidelines.xhtml

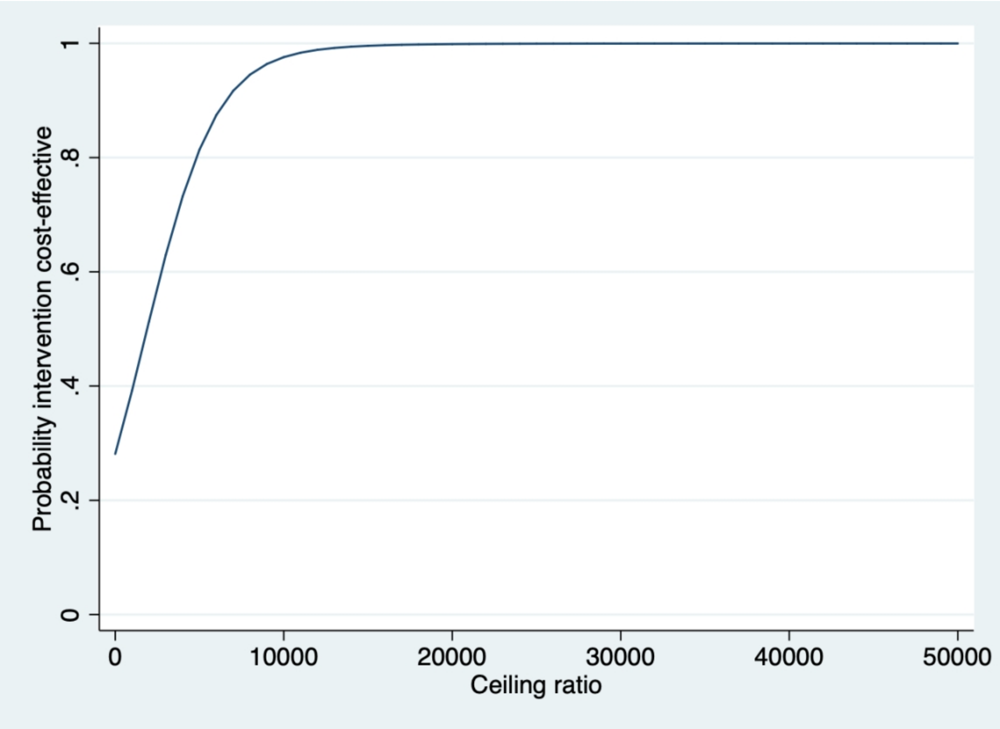

Supplementary Figure 2. Main analysis – societal perspective. Cost-effectiveness acceptability curve for clinically relevant response (improvement of ≥ 3 points on Vertigo Symptom Scale - Short Form) comparing stand-alone VR with usual care.

VR = vestibular rehabilitation

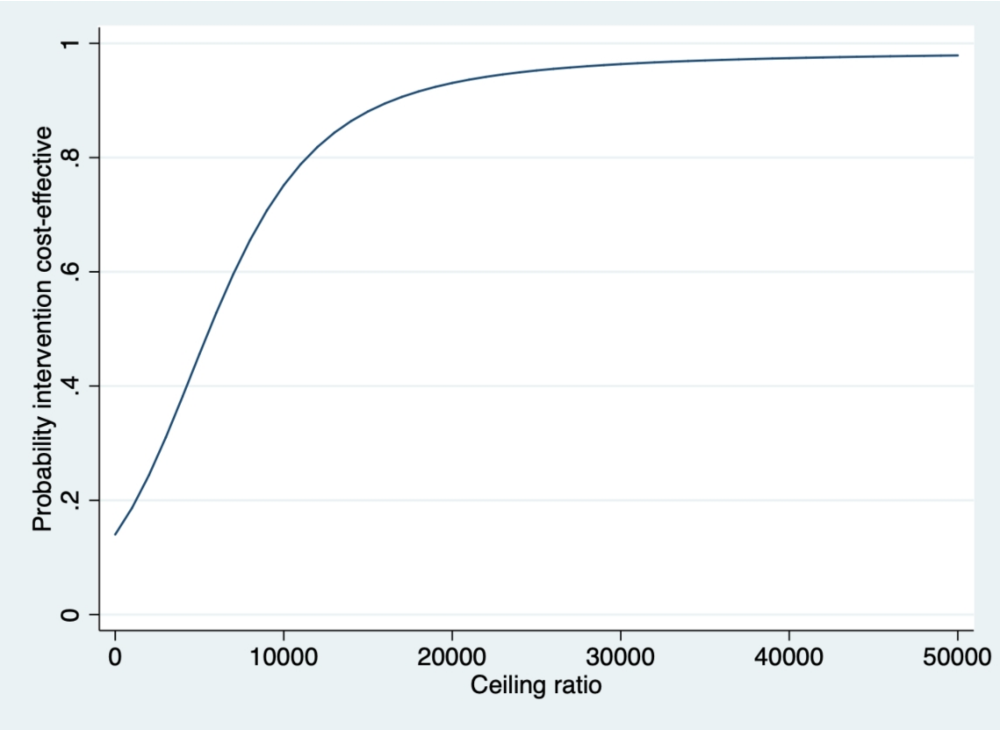

Supplementary Figure 3. Main analysis – societal perspective. Cost-effectiveness acceptability curve for clinically relevant response (improvement of ≥ 3 points on Vertigo Symptom Scale - Short Form) comparing blended VR with usual care.

VR = vestibular rehabilitation

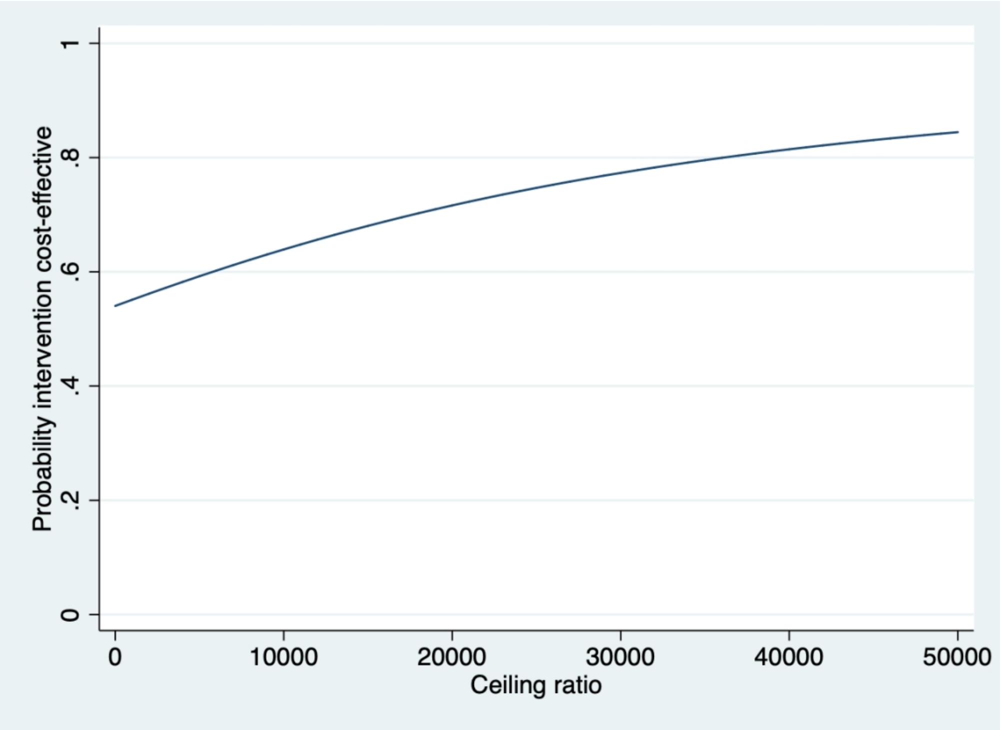

Supplementary Figure 4. SA3 – societal perspective. Cost-effectiveness acceptability curve for Quality-Adjusted Life Years comparing stand-alone VR with usual care.

SA3 = sensitivity analysis with secondary care and medication costs excluded
VR = vestibular rehabilitation

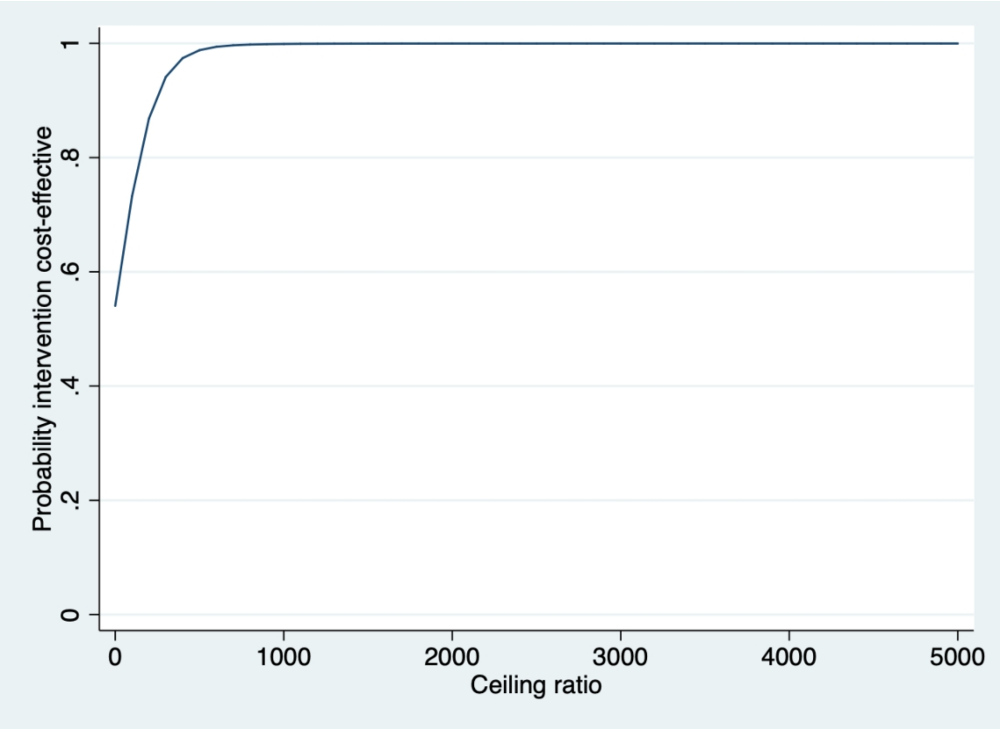

Supplementary Figure 5. SA3 – societal perspective. Cost-effectiveness acceptability curve for the Vertigo Symptom Scale - Short Form comparing stand-alone VR with usual care.

SA3 = sensitivity analysis with secondary care and medication costs excluded
VR = vestibular rehabilitation

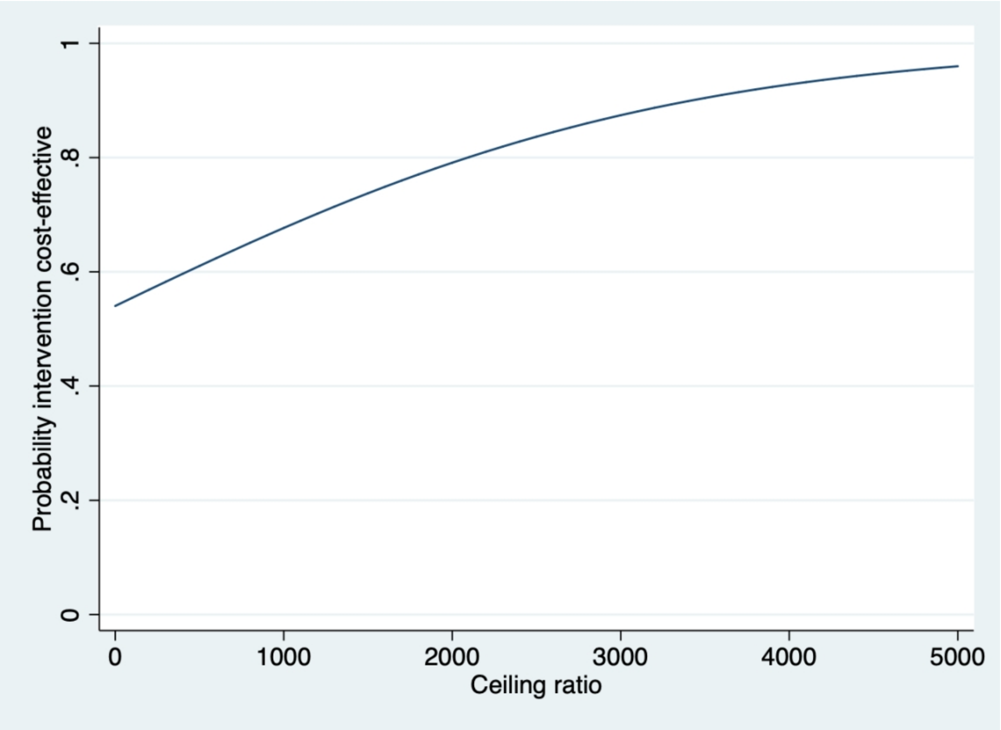

Supplementary Figure 6. SA3 – societal perspective. Cost-effectiveness acceptability curve for clinically relevant response (improvement of ≥ 3 points on Vertigo Symptom Scale - Short Form) comparing stand-alone VR with usual care.SA3 = sensitivity analysis with secondary care and medication costs excludedVR = vestibular rehabilitation

Supplementary Table 1 Baseline characteristics. Figures are numbers (percentages) unless stated otherwise.

		Stand-alone VR (N=98)	Blended VR (N=104)	Usual care (N=120)	Total sample (N=322)
Mean (SD) age (years)		66.7 (9.5)	67.4 (9.8)	67.0 (9.4)	67.0 (9.5)
Female		64 (65)	69 (66)	64 (53)	197 (61)
Level of education					
	Low	33 (34)	37 (36)	36 (30)	106 (33)
	Middle	25 (26)	31 (30)	30 (25)	86 (27)
	High	40 (41)	36 (35)	54 (45)	130 (40)
Living situation					
	Alone	34 (35)	33 (32)	35 (29)	102 (32)
	With partner	64 (65)	71 (68)	85 (71)	220 (68)
Number of chronic diseases*					
	0	59 (60)	64 (62)	63 (53)	186 (58)
	1	28 (29)	32 (31)	41 (34)	101 (31)
	2	8 (8)	4 (4)	12 (10)	24 (7)
	≥3	3 (3)	4 (4)	4 (3)	11 (3)
Time since vestibular diagnosis**					
	One to six months	15 (15)	22 (21)	13 (11)	50 (16)
	Six months to two years	28 (29)	27 (26)	39 (33)	94 (29)
	Two years to 10 years	31 (32)	44 (42)	48 (40)	123 (38)
	More than 10 years	23 (24)	11 (11)	18 (15)	52 (16)
Self-reported vestibular diagnosis**					
	No known diagnosis	67 (68)	69 (66)	77 (64)	213 (66)
	Benign paroxysmal positional vertigo	11 (11)	17 (16)	22 (18)	50 (16)
	Meniere's disease	9 (9)	9 (9)	10 (8)	28 (9)
	Vestibular neuritis	6 (6)	4 (4)	7 (6)	17 (5)
	PPPD	0 (0)	1 (1)	0 (0)	1 (0)
	Other^	4 (4)	4 (4)	2 (2)	10 (3)
Disorders at baseline according to PHQ		14 (14)	16 (15)	23 (19)	53 (17)
	Panic disorder	2 (2)	3 (3)	5 (4)	10 (3)
	Generalised anxiety disorder	12 (12)	15 (14)	19 (16)	46 (14
	Major depressive disorder	5 (5)	6 (6)	9 (8)	20 (6)

PPPD = persistent postural-perceptual dizziness; PHQ = patient health questionnaire.
*Chronic non-specific lung disease, cardiac disease, peripheral arterial disease, stroke, diabetes mellitus, arthritis, and cancer.
**Data on this variable was missing for three participants: stand-alone VR (n=1), usual care (n=2).
^ Traumatic brain injury, cerebrovascular accident, Parkinson's disease, bacterial meningitis, and vestibular organ surgical procedures.

For peer review only

Supplementary Table 2 Mean costs (SE) stratified for treatment group after recoding outliers as missing (SA2) and cost outcomes (95% CI) for the intervention groups compared to usual care.

Cost category	Stand-alone VR N=98	Blended VR N=104	Usual care N=120	Difference stand-alone VR versus usual care (95% CI)	Difference blended VR versus usual care (95% CI)
Healthcare costs	846 (118)	1428 (232)	775 (103)	71 (-221 ; 368)	653 (-254 ; 1257)
Lost productivity costs	2465 (684)	2992 (766)	2083 (462)	383 (-998 ; 2199)	910 (-505 ; 3026)
Total societal costs	3311 (736)	4420 (825)	2857 (502)	454 (-1019 ; 2399)	1563 (-47 ; 3764)

SE = standard error; VR = vestibular rehabilitation; CI = confidence interval

For peer review only

CHEERS checklist—Items to include when reporting economic evaluations of health interventions

Section/item	Item No	Recommendation	Reported on page No/ line No
**Title and abstract**			
Title	1	Identify the study as an economic evaluation or use more specific terms such as "cost-effectiveness analysis", and describe the interventions compared.	Page 1, line 1 to 3
Abstract	2	Provide a structured summary of objectives, perspective, setting, methods (including study design and inputs), results (including base case and uncertainty analyses), and conclusions.	Page 2, line 32 to page 3, line 64
**Introduction**			
Background and objectives	3	Provide an explicit statement of the broader context for the study.	Page 4, line 77 to 83
		Present the study question and its relevance for health policy or practice decisions.	Page 5, line 114 to 116
**Methods**			
Target population and subgroups	4	Describe characteristics of the base case population and subgroups analysed, including why they were chosen.	Page 6, line 128 to 137
Setting and location	5	State relevant aspects of the system(s) in which the decision(s) need(s) to be made.	Page 6, line 119 to 130
Study perspective	6	Describe the perspective of the study and relate this to the costs being evaluated.	Page 9, line 203 to 210
Comparators	7	Describe the interventions or strategies being compared and state why they were chosen.	Page 6, line 139 to Page 7, line 161
Time horizon	8	State the time horizon(s) over which costs and consequences are being evaluated and say why appropriate.	Page 9, line 203 to 210
Discount rate	9	Report the choice of discount rate(s) used for costs and outcomes and say why appropriate.	Page 9, line 203 to 210
Choice of health outcomes	10	Describe what outcomes were used as the measure(s) of benefit in the evaluation and their relevance for the type of analysis performed.	Page 7, line 163 to Page 9, line 201
Measurement of effectiveness	11a	*Single study-based estimates:* Describe fully the design features of the single effectiveness study and why the single study was a sufficient source of clinical effectiveness data.	Page 6, line 119 to Page 7, line 161
	11b	*Synthesis-based estimates*: Describe fully the methods used for identification of included studies and synthesis of clinical effectiveness data.	Not applicable
Measurement and valuation of preference based outcomes	12	If applicable, describe the population and methods used to elicit preferences for outcomes.	Not applicable
Estimating resources and costs	13a	*Single study-based economic evaluation:* Describe approaches used to estimate resource use associated with the alternative interventions. Describe primary or secondary research methods for valuing each resource item in terms of its unit cost. Describe any adjustments made to approximate to opportunity costs.	Page 8, line 179 to Page 9, line 201
	13b	*Model-based economic evaluation:* Describe approaches and data sources used to estimate	Not applicable

For peer review only

Section/item	Item No	Recommendation	Reported on page No/ line No
		resource use associated with model health states. Describe primary or secondary research methods for valuing each resource item in terms of its unit cost. Describe any adjustments made to approximate to opportunity costs.	
Currency, price date, and conversion	14	Report the dates of the estimated resource quantities and unit costs. Describe methods for adjusting estimated unit costs to the year of reported costs if necessary. Describe methods for converting costs into a common currency base and the exchange rate.	Page 8, line 179 to Page 9, line 201
Choice of model	15	Describe and give reasons for the specific type of decision-analytical model used. Providing a figure to show model structure is strongly recommended.	Page 9, line 203 to page 10, line 234
Assumptions	16	Describe all structural or other assumptions underpinning the decision-analytical model.	Page 9, line 203 to page 10, line 234
Analytical methods	17	Describe all analytical methods supporting the evaluation. This could include methods for dealing with skewed, missing, or censored data; extrapolation methods; methods for pooling data; approaches to validate or make adjustments (such as half cycle corrections) to a model; and methods for handling population heterogeneity and uncertainty.	Page 9, line 203 to page 10, line 234
**Results**			
Study parameters	18	Report the values, ranges, references, and, if used, probability distributions for all parameters. Report reasons or sources for distributions used to represent uncertainty where appropriate. Providing a table to show the input values is strongly recommended.	Page 12, line 256 to page 15, line 346 Table 1, Table 2 and Table 3
Incremental costs and outcomes	19	For each intervention, report mean values for the main categories of estimated costs and outcomes of interest, as well as mean differences between the comparator groups. If applicable, report incremental cost-effectiveness ratios.	Page 12, line 256 to page 15, line 346 Table 1, Table 2 and Table 3
Characterising uncertainty	20a	*Single study-based economic evaluation:* Describe the effects of sampling uncertainty for the estimated incremental cost and incremental effectiveness parameters, together with the impact of methodological assumptions (such as discount rate, study perspective).	Page 12, line 256 to page 15, line 346 Table 1, Table 2 and Table 3
	20b	*Model-based economic evaluation:* Describe the effects on the results of uncertainty for all input parameters, and uncertainty related to the structure of the model and assumptions.	Not applicable
Characterising heterogeneity	21	If applicable, report differences in costs, outcomes, or cost-effectiveness that can be explained by variations between subgroups of patients with different baseline characteristics or other observed variability in effects that are not reducible by more information.	Not applicable
**Discussion**			
Study findings, limitations, generalisability, and current knowledge	22	Summarise key study findings and describe how they support the conclusions reached. Discuss limitations and the generalisability of the findings and how the findings fit with current knowledge.	Page 16, line 348 to Page 19, line 425
**Other**			
Source of funding	23	Describe how the study was funded and the role of	Page 20, line 436 to 440

For peer review only

Section/item	Item No	Recommendation	Reported on page No/ line No
		the funder in the identification, design, conduct, and reporting of the analysis. Describe other non-monetary sources of support.	
Conflicts of interest	24	Describe any potential for conflict of interest of study contributors in accordance with journal policy. In the absence of a journal policy, we recommend authors comply with International Committee of Medical Journal Editors recommendations.	Page 20, line 429 to 434

For consistency, the CHEERS statement checklist format is based on the format of the CONSORT statement checklist

For peer review only

BMJ Open

The cost-effectiveness of internet-based vestibular rehabilitation with and without physiotherapy support for adults aged 50 and older with a chronic vestibular syndrome in general practice

Journal:	*BMJ Open*
Manuscript ID	bmjopen-2019-035583.R1
Article Type:	Original research
Date Submitted by the Author:	25-Jun-2020

Complete List of Authors:	van Vugt, Vincent; Amsterdam UMC - Locatie VUMC, Department of general practice; Amsterdam Public Health research institute<br>Bosmans, J; Vrije Universiteit Amsterdam, Department of Health Sciences; Amsterdam Public Health research institute<br>Finch, Aureliano; Vrije Universiteit Amsterdam, Department of Health Sciences; Amsterdam Public Health research institute<br>van der Wouden, Johannes; Amsterdam UMC - Locatie VUMC, Department of general practice; Amsterdam Public Health research institute<br>van der Horst, Henriette; Amsterdam UMC - Locatie VUMC, Department of general practice; Amsterdam Public Health research institute<br>Maarsingh, Otto; Amsterdam UMC - Locatie VUMC, Department of general practice; Amsterdam Public Health research institute

**Primary Subject Heading**:	General practice / Family practice
Secondary Subject Heading:	Neurology, Health economics
Keywords:	Health economics < HEALTH SERVICES ADMINISTRATION & MANAGEMENT, PRIMARY CARE, REHABILITATION MEDICINE, NEUROLOGY, OTOLARYNGOLOGY

SCHOLARONE™
Manuscripts

I, the Submitting Author has the right to grant and does grant on behalf of all authors of the Work (as defined in the below author licence), an exclusive licence and/or a non-exclusive licence for contributions from authors who are: i) UK Crown employees; ii) where BMJ has agreed a CC-BY licence shall apply, and/or iii) in accordance with the terms applicable for US Federal Government officers or employees acting as part of their official duties; on a worldwide, perpetual, irrevocable, royalty-free basis to BMJ Publishing Group Ltd ("BMJ") its licensees and where the relevant Journal is co-owned by BMJ to the co-owners of the Journal, to publish the Work in this journal and any other BMJ products and to exploit all rights, as set out in our licence.

The Submitting Author accepts and understands that any supply made under these terms is made by BMJ to the Submitting Author unless you are acting as an employee on behalf of your employer or a postgraduate student of an affiliated institution which is paying any applicable article publishing charge ("APC") for Open Access articles. Where the Submitting Author wishes to make the Work available on an Open Access basis (and intends to pay the relevant APC), the terms of reuse of such Open Access shall be governed by a Creative Commons licence – details of these licences and which Creative Commons licence will apply to this Work are set out in our licence referred to above.

Other than as permitted in any relevant BMJ Author's Self Archiving Policies, I confirm this Work has not been accepted for publication elsewhere, is not being considered for publication elsewhere and does not duplicate material already published. I confirm all authors consent to publication of this Work and authorise the granting of this licence.

For peer review only

The cost-effectiveness of internet-based vestibular rehabilitation with and without physiotherapy support for adults aged 50 and older with a chronic vestibular syndrome in general practice

Vincent A. van Vugt[1], Judith E. Bosmans[2], Aureliano P. Finch[2], Johannes C. van der Wouden[1], Henriëtte E. van der Horst[1], Otto R. Maarsingh[1]

[1] Amsterdam UMC, Vrije Universiteit Amsterdam, Department of general practice and elderly care medicine, Amsterdam Public Health, de Boelelaan 1117, Amsterdam, Netherlands

[2] Department of Health Sciences, Faculty of Science, Amsterdam Public Health Research Institute, Vrije Universiteit Amsterdam, Amsterdam, Netherlands.

Corresponding author:
Vincent A. van Vugt, MD
Amsterdam UMC - Location VUMC
Department of general practice and elderly care medicine
Van der Boechorststraat 7, room C-378. 1081 BT Amsterdam, The Netherlands
Phone: +31613275226
Email: v.vanvugt@amsterdamumc.nl
ORCID number: 0000-0002-2746-4820

Number of Tables: 3
Number of Figures: 2
Number of Supplementary Tables/Figures: 8
Keywords: general practice, primary care, dizziness, vertigo, vestibular rehabilitation, cost-effectiveness, economic evaluation, chronic vestibular syndrome
Pages: 22
Word count: 3865
Disclosures: none

For peer review only

ABSTRACT

Objectives To evaluate the cost-effectiveness of stand-alone and blended internet-based vestibular rehabilitation (VR) in comparison with usual care (UC) for chronic vestibular syndromes in general practice.

Design Economic evaluation alongside a three-armed, individually randomised controlled trial.

Setting 59 Dutch general practices.

Participants 322 adults, aged 50 years and older with a chronic vestibular syndrome.

Interventions Stand-alone VR consisted of a six-week, internet-based intervention with weekly online sessions and daily exercises. In blended VR, this intervention was supplemented with face-to-face physiotherapy support. UC group participants received usual general practice care without restrictions.

Main outcome measures Societal costs, quality-adjusted life years (QALY), Vertigo Symptom Scale - Short Form (VSS-SF), clinically relevant response (≥3 points VSS-SF improvement).

Results Mean societal costs in both the stand-alone and blended VR groups were statistically non-significantly higher than in the UC group (mean difference (MD) €504, 95%-CI -1082 to 2268; and €916, 95%-CI -663 to 2596). Both stand-alone and blended VR groups reported non-significantly more QALYs than the UC group (MD 0.02 , 95%-CI -0.00 to 0.04; and 0.01, 95%-CI -0.01 to 0.03), and significantly better VSS-SF scores (MD 3.8 points, 95%-CI 1.7 to 6.0; and 3.3 points, 95%-CI 1.3 to 5.2). For stand-alone VR compared to UC, the probability of cost-effectiveness was 0.95 at a willingness-to-pay ratio of 24,161 €/QALY, 600 €/point improvement in VSS-SF, and 8000 €/clinically relevant responder in VSS-SF. For blended VR versus UC, the probability of cost-effectiveness was 0.95 at a willingness-to-pay

For peer review only - http://bmjopen.bmj.com/site/about/guidelines.xhtml

ratio of 123,335 €/QALY, 900 €/point improvement in VSS-SF and 24,000 €/clinically relevant responder in VSS-SF.

Conclusion Stand-alone and blended internet-based VR non-significantly increased QALYs and significantly reduced vestibular symptoms compared to UC, while costs in both groups were non-significantly higher. Stand-alone VR has the highest probability to be cost-effective compared to UC.

Trial registration Netherlands Trial Register NTR5712

Strengths and limitations of this study

- This is the first study to evaluate the cost-effectiveness of stand-alone internet-based vestibular rehabilitation and blended vestibular rehabilitation versus usual care for patients with a chronic vestibular syndrome in general practice.

- The economic evaluation was conducted based on the data of 322 participants aged 50 years and older with a chronic vestibular syndrome.

- Strengths of our study are the societal perspective on costs, the use of multiple outcome measures and the use of a sensitivity analysis to test robustness of our findings.

- Limitations of the study are that it was powered for the primary outcome measure VSS-SF and not for costs or QALY's, and that the follow-up was limited to six months.

For peer review only

INTRODUCTION

General practitioners frequently encounter patients with vestibular symptoms (i.e. vertigo, dizziness, vestibulovisual and postural symptoms).[1-3] The one-year prevalence of vestibular symptoms is approximately 20% in population-based studies.[1] Patients with vestibular symptoms are often unable to work, make frequent use of healthcare services and have an increased risk of falling.[1, 4, 5] The financial burden of these symptoms is therefore substantial. In a recent German study, the yearly healthcare costs were over 800 euro higher in patients with vestibular symptoms than in patients without vestibular symptoms.[5] Through assessment of timing and triggers of symptoms, patients can be classified as having either an acute vestibular syndrome, episodic vestibular syndrome or chronic vestibular syndrome.[6-8] In chronic vestibular syndromes, patients experience vestibular symptoms with features suggestive of persistent vestibular system dysfunction for months to years. The preferred treatment for chronic vestibular syndromes, according to clinical guidelines from the United States,[9, 10] the United Kingdom[11] and The Netherlands,[12] is vestibular rehabilitation (VR). VR is an exercise-based treatment developed to reduce vestibular symptoms by gradually stimulating the vestibular system.[13, 14] There is moderate to strong evidence that VR can safely and effectively reduce vestibular symptoms in both unilateral and bilateral peripheral vestibular dysfunction. [14, 15]

Despite the scientific evidence and recommendations in guidelines, VR is still underused in general practice. Surveys among Dutch [16] and English [17] general practitioners (GPs) indicate that less than 10% uses VR. Developing new ways to deliver VR in general practice may help to implement this effective treatment in daily practice. Internet interventions have several advantages over other forms of treatment: they are inexpensive, easily accessible and can be easily personalised according to the needs of individual patients. Recently, the

For peer review only - http://bmjopen.bmj.com/site/about/guidelines.xhtml

University of Southampton transformed the content of a VR booklet[18] that was shown to be (cost-)effective compared to usual care, into an internet-based VR intervention.[19] In a randomised controlled trial in the UK,[20] this stand-alone internet-based VR intervention effectively reduced vestibular symptoms compared to usual care. Combining an internet-based intervention with face-to-face support by a healthcare professional is called blended care.[21] Stand-alone internet-based interventions are generally less expensive than blended internet-based interventions, but the risk of non-adherence is also higher.[21-23] We therefore developed a blended internet-based VR intervention by adding physiotherapy support to the British internet-based VR intervention. We conducted a pragmatic, three-armed, randomised controlled trial in Dutch general practice to investigate the clinical effectiveness and cost-effectiveness of stand-alone and blended internet-based VR compared to usual care.[24] As we recently reported, both stand-alone and blended internet-based vestibular rehabilitation led to a clinically relevant and statistically significant decrease in vestibular symptoms compared to usual care.[25] The objective of the present substudy is to evaluate the cost-effectiveness of stand-alone and blended internet-based VR versus usual care for general practice patients aged 50 and older with a chronic vestibular syndrome.

For peer review only

For peer review only - http://bmjopen.bmj.com/site/about/guidelines.xhtml

METHODS

Design

This economic evaluation was conducted alongside a pragmatic, three-armed, randomised controlled trial in general practice. In the trial, we compared stand-alone and blended internet-based VR with usual care in patients aged 50 and older with a chronic vestibular syndrome. A detailed description of the study protocol(24) and the results of the clinical effectiveness analysis(25) can be found in previous publications. For the reporting of the cost-effectiveness analysis, we follow the recommendations in the Consolidated Health Economic Evaluation Reporting Standards (CHEERS) statement.(26)

Participants

In short, we recruited participants from 59 general practices in The Netherlands. Patients with chronic vestibular syndrome according to the International Classification of Vestibular Disorders (ICVD) were eligible for the study.(6, 7) Chronic vestibular syndrome was defined as vestibular symptoms at time of inclusion that had been present for at least one month and that were exacerbated or triggered by performing head movement. Further inclusion criteria were: age 50 years and older; good command of the Dutch language; access to the internet and an email account. Participants with an identified non-vestibular cause of dizziness, medical contraindications for making the required head movements (e.g. severe cervical arthrosis), serious comorbid conditions precluding participation in an exercise programme, or current enrolment in another study were excluded.

Interventions

Stand-alone internet-based vestibular rehabilitation (stand-alone VR)

For peer review only - http://bmjopen.bmj.com/site/about/guidelines.xhtml

Vertigo Training, the internet-based VR intervention we used in this trial, is a Dutch translation of the internet-based VR intervention developed by the University of Southampton. The intervention lasted six weeks and consisted of six weekly online sessions with daily VR exercises. In the first session, video demonstrations and written instructions are used to teach participants the six core VR exercises. During the intervention period, participants were asked to perform these exercises for 10 minutes twice daily. Every week, the participant logged in to Vertigo Training to self-report the level of vestibular symptoms caused by each of the six exercises. Vertigo Training used this information to produce a VR exercise prescription for the coming week, tailored to the individual needs and capabilities of the participant. In addition, Vertigo Training also provides information and advice on coping and symptom control strategies which are described in more detail elsewhere.[25]

Blended internet-based vestibular rehabilitation with physiotherapist support (blended VR)

Supplemental to the Vertigo Training intervention, participants in the blended VR group were visited twice at home by a trained physiotherapist. These supportive physiotherapy sessions occurred in weeks one and three of the six-week intervention period and lasted for 45 minutes each.

Usual care

Participants in the usual care group received the standard level of care provided by their own GP without restrictions.

Measures

We assessed quality of life with the most widely used preference-based quality-of-life instrument in economic evaluations, [27] the 5-level EuroQol questionnaire (EQ-5D-5L), at

For peer review only - http://bmjopen.bmj.com/site/about/guidelines.xhtml

baseline, and 3 and 6 months of follow-up.[28] EQ-5D-5L health states were converted to utility scores using the Dutch EQ-5D-5L tariff. [29] For all five dimensions, a subtraction is done from the utility score if the participant experiences any problems on that domain. We calculated Quality-Adjusted Life-Years (QALYs) by multiplying the utility of a specific health state with the time spent in that health state. Transitions between health states were linearly interpolated. One QALY is equivalent to one life-year in perfect health. A gain in QALYs can therefore be interpreted as an improvement in the quantity and/or quality of life.

We measured vestibular symptoms by the Vertigo Symptom Scale-Short Form (VSS-SF)[30, 31] at baseline, and 3 and 6 months of follow-up. The VSS-SF measures the frequency of 15 vestibular symptoms on a scale from 0 (no symptoms) to 4 (symptoms most days) during the past month (total range 0-60 points). Improvement can reflect either fewer or less frequent symptoms. In accordance with previous studies,[18, 20, 32, 33] we defined a decrease of three points or more on the VSS-SF between baseline and six month measurement as a clinically relevant response. In this cost-effectiveness study, we used the VSS-SF scores at 6 months follow-up and the number of participants with a clinically relevant improvement during the 6-month trial period as measures of effectiveness.

We measured costs from a societal perspective, using the iMTA Medical and Productivity Cost Questionnaires at 3 and 6 months of follow-up.[34, 35] Both questionnaires had a recall period of 3 months. The societal costs included healthcare costs (primary care, secondary care, medication and home care), informal care costs (i.e. costs related to help from family and friends) and lost productivity costs. Lost productivity costs consisted of costs related to absenteeism from paid and unpaid work (e.g. household activities or voluntary work), and

For peer review only - http://bmjopen.bmj.com/site/about/guidelines.xhtml

presenteeism costs. Presenteeism is defined as reduced efficiency due to health problems while at work.[36]

We valued healthcare utilisation by using Dutch standard costs if available.[37] Otherwise, we used tariffs of professional organisations or healthcare providers themselves. We used data from the Dutch Healthcare Institute to value medication use.[38] We estimated costs of absenteeism from paid work by using the friction cost approach[39] that assumes that sick employees are replaced after a certain period of time (the friction period, i.e. 12 weeks) after which there are no productivity losses anymore. We calculated productivity losses associated with paid work with gender-specific average wage rates of the Dutch population.[37] We assessed presenteeism by measuring participants' efficiency while at work on a numerical rating scale ranging from 0 ("I could not do anything") to 10 ("I did as much as always", i.e. the efficiency score. To calculate lost productivity hours due to presenteeism, we multiplied one minus the efficiency score with the number of days of reduced efficiency, multiplied by the number of working hours per day. We then used gender-specific average wage rates to convert the lost productivity hours due to presenteeism to lost productivity costs. To calculate lost productivity costs associated with unpaid work and informal care costs, we used a shadow price for a legally employed cleaner.[37]

For peer review only

Cost-effectiveness analyses

We performed the economic evaluation from a societal perspective. We compared QALYs and VSS-SF scores at 6 months pairwise between the two active treatment groups (stand-alone and blended VR) and usual care. Discounting was not necessary, because follow-up was shorter than 1 year.

For peer review only - http://bmjopen.bmj.com/site/about/guidelines.xhtml

We performed the analyses according to the intention to treat principle. We imputed missing cost and effect data with multiple imputation by chained equations (MICE) with predictive mean matching to account for the skewed distribution of costs.(40) The advantage of multiple imputation over single imputation methods is that the uncertainty around the imputed values is also taken into account. The predictive mean matching ensured that only observed values can be imputed. The number of imputed datasets was increased until the fraction of missing information was smaller than 5%.(41) The imputation model included variables that differed at baseline, differed between participants with and without complete follow-up, or that were associated with the outcomes (clinical effects and costs). In addition, we included all variables of the analysis models in the imputation model. We performed analyses separately for each imputed dataset and then pooled applying Rubin's rules.(42)

We estimated differences in costs and effects with bivariate regression in which two separate regression models are specified, while correlation between costs and effects is maintained through correlated error terms. This allows for specification of separate covariates for costs and effects in the regression models. We used bias-corrected accelerated bootstrapping with 5,000 replications to estimate the uncertainty surrounding differences in costs and effects. We calculated incremental cost-effectiveness ratios (ICERs) by dividing the difference in costs by the difference in effects. ICERs indicate the investment that is needed to gain one unit of effect extra in the intervention group compared to control. Uncertainty surrounding the ICER is shown in cost-effectiveness (CE) planes.(43) The probability of cost-effectiveness was calculated by determining the proportion of bootstrapped cost-effect pairs below the willingness-to-pay threshold for each possible willingness-to-pay threshold. By estimating cost-effectiveness acceptability (CEA), we subsequently combined statistical uncertainty with decision uncertainty curves to show the probability that the intervention is cost-effective

For peer review only - http://bmjopen.bmj.com/site/about/guidelines.xhtml

compared to usual care for different willingness-to-pay ratios.[44] The willingness-to-pay ratio is defined as the maximum amount of money that society is willing to pay to gain one additional unit of effect. We employed the commonly used threshold of 20,000 € per QALY gained for appraisal of new health technologies. [45]

We conducted three sensitivity analyses to assess the robustness of the findings. In the first sensitivity analysis (SA1), we conducted the economic evaluation from the perspective of the healthcare system, meaning that only healthcare costs are included, as is recommended in some other countries, such as the UK, Belgium and Germany. In the second sensitivity analysis (SA2), we coded costs of extreme outliers (five participants who were admitted to the intensive care unit during the trial for reasons unrelated to the trial) as missing so they would be imputed in the multiple imputation procedure. In the third sensitivity analysis (SA3), we excluded secondary care and medication costs. We chose to conduct SA3 because hospitalisations and expensive medications could have had a large influence on the main analysis due to relatively high costs, even though they were all judged to be unrelated to vestibular symptoms.

Patient and public involvement

Patients played an important role in the development of Vertigo Training. Detailed feedback by patients with vestibular symptoms on the content, usability and Dutch translation in prototype versions led to some amendments of the online intervention. No patients advised on interpretation of the results, nor were they involved in writing the manuscript. A lay summary of the research findings will be distributed to all participants in the study and the results will be disseminated to the relevant patient community.

For peer review only - http://bmjopen.bmj.com/site/about/guidelines.xhtml

RESULTS

Participants

We provide an overview of patient enrolment, allocation and follow-up in Supplementary Figure 1. Participants were recruited between June 2017 and July 2018. We randomised 322 participants at baseline: 98 participants were allocated to the stand-alone VR group, 104 to the blended VR group, and 120 to the usual care group. The baseline characteristics of participants are shown in Supplementary Table 1.

Effects

Table 1 presents the pooled mean effects and costs in the three intervention groups. The mean QALY was 0.43 for stand-alone VR participants, 0.41 for blended VR participants and 0.41 for usual care participants. The differences of stand-alone and blended VR compared to usual care in QALYs were not statistically significant (mean difference 0.02 , 95% CI -0.00 to 0.04; and 0.01, 95% CI -0.01 to 0.03). Both stand-alone and blended VR participants reported statistically significantly lower VSS-SF scores at 6 months follow-up compared to usual care participants (mean difference 3.8 points, 95% CI 1.7 to 6.0; and 3.3 points, 95% CI 1.3 to 5.2). A clinically relevant VSS-SF response was seen significantly more often in both stand-alone and blended VR groups than in the usual care group (difference in percentage of participants with clinically relevant response 27%, 95% CI 13% to 40%; and 16%, 95% CI 2% to 30%). Overall, differences in effects for stand-alone VR versus usual care were larger than for blended VR versus usual care.

Costs

Total societal costs in the stand-alone group were €3567 (standard error 701), in the blended VR group €3979 (SE 667), and in the usual care group €3063 (SE 520). However, uncertainty

For peer review only - http://bmjopen.bmj.com/site/about/guidelines.xhtml

For peer review only

surrounding these differences was substantial. The cost difference between stand-alone VR and usual care was smaller (i.e. mean difference €504, 95% CI -1082 to 2268) than between blended VR and usual care (mean difference €916, 95% CI -663 to 2596). Healthcare costs in both the stand-alone and blended VR groups were statistically non-significantly higher than in the usual care group (mean difference €478, 95% CI -100 to 1514; and €215, 95% CI -272 to 836). Total lost productivity costs in the stand-alone VR and usual care groups were similar (mean difference €12, 95% CI -1260 to 1439). Total lost productivity costs in the blended VR group were considerably – but not significantly – higher than in the usual care group (mean difference €472, 95% CI -846 to 1825).

Cost-effectiveness main analysis

Table 2 presents the results of the cost-effectiveness analyses for stand-alone VR compared with usual care. For QALYs, the ICER was 24,161 €/QALY gained for stand-alone VR in comparison with usual care. The probability of cost-effectiveness was 0.28 at a willingness-to-pay ratio of 0 €/QALY gained. At a willingness-to-pay ratio of 20,000 €/QALY gained the probability of cost-effectiveness was 0.47 for stand-alone VR versus usual care. For VSS-SF, the ICER was 132, indicating that €132 needs to be invested to gain one point of improvement in VSS-SF in the stand-alone VR group compared with usual care. The probability that stand-alone VR is cost-effective compared to usual care was 0.28 and 0.95 at willingness-to-pay ratios of 0 and 600 €/point improvement in VSS-SF, respectively (Figure 1). The ICER for response indicates that one additional participant with a clinically relevant response on the VSS-SF (≥3 points improvement) requires an investment of on average €1895 in the stand-alone VR group compared to the usual care group. At ceiling ratios of 0 and 8000 €/additional responder, the probability that stand-alone care is cost-effective in comparison with usual care was 0.28 and 0.95, respectively (Supplementary Figure 2).

For peer review only - http://bmjopen.bmj.com/site/about/guidelines.xhtml

In Table 3 the results of the cost-effectiveness analyses for blended VR in comparison with usual care are shown. For QALYs, the ICER was 123,335 (the large ICER is caused by the small difference in effects), meaning that €123,335 needs to be invested to gain 1 QALY in the blended VR group compared to the usual care group. The probability of cost-effectiveness was 0.14 at a ceiling ratio of 0 €/QALY gained and increased to 0.21 at a ceiling ratio of 20,000 €/QALY gained. For VSS-SF, the ICER was 280 for blended VR compared with usual care, indicating that to gain one point of improvement in VSS-SF, €280 needs to be invested in blended VR compared with usual care. The CEA curve (Figure 2) shows that the probability of cost-effectiveness rapidly increases from 0.14 at a willingness-to-pay ratio of 0 €/point improvement in VSS-SF to 0.95 at a willingness-to-pay ratio of 900 €/point of improvement in VSS-SF. The ICER for response was 5599, indicating that one patient extra with a clinically relevant response on the VSS-SF (≥3 points improvement), is associated with an investment of €5599 for blended VR in comparison with usual care. The CEA curve (Supplementary Figure 3) indicates that the probability of cost-effectiveness is 0.14 and 0.95 at ceiling ratios of 0 and 24,000 €/responder extra, respectively.

Sensitivity analyses

Table 2 presents the results of the sensitivity analyses for stand-alone VR compared with usual care and Table 3 shows the results of the sensitivity analyses for blended VR compared with usual care. In SA1, in which the analysis was performed from the healthcare perspective, healthcare costs in the stand-alone VR group were higher than in the usual care group (mean difference €478, 95% CI -100 to 1514), although the differences in healthcare costs were smaller than the differences in societal costs (mean difference €504, 95% CI -1064 to 2303). There was less statistical uncertainty surrounding healthcare costs compared to societal costs.

For peer review only - http://bmjopen.bmj.com/site/about/guidelines.xhtml

In this sensitivity analysis, the ICERs for stand-alone VR compared to usual care with regard to QALYs (22,936), VSS-SF (126) and response (1799) were all slightly lower than in the main analysis (QALYs 24,161; VSS-SF 132; and response 1895). Healthcare costs in the blended VR group were higher than in the usual care group, but smaller than the difference in societal costs in the main analysis (mean difference €215, 95% CI -272 to 836; versus €916, 95% CI -663 to 2596). As a result, in the comparison between blended VR and usual care, the ICERS for QALY's (28,848), VSS-SF (65) and response (1310) were substantially lower than in the main analysis (QALYs 123,335; VSS-SF 280; and response 5599).

In SA2, where we coded the extreme outliers as missing and imputed their data, the societal cost difference between stand-alone VR and usual care decreased from €504 (95% CI -1082 to 2268) to €454 (95% CI -1019 to 2399), as shown in Supplementary Table 2. For stand-alone VR in comparison to usual care, the ICERS for QALY's, VSS-SF and response were all markedly lower and the probability of cost-effectiveness at a willingness-to-pay ratio of 0 €/unit of effect extra increased from 0.28 in the main analysis to 0.39 in SA2 (Table 2). For blended VR compared with usual care, the societal cost difference became larger and the probabilities of cost-effectiveness lower (Table 3).

In SA3, where we excluded costs not related to vestibular symptoms, results become more positive due to smaller differences in costs between the stand-alone and blended VR groups and usual care. For blended VR in comparison with usual care, this effect is relatively small (cost difference €810, 95% CI -653 to 2400; instead of €916, 95% CI -663 to 2596), resulting in similar probabilities of cost-effectiveness as in the main analysis (Table 3). However, for stand-alone VR in comparison with usual care the cost difference inverts (Table 2; -€75, 95% CI -1425 to 1450; instead of €504, 95% CI -1064 to 2303). As a result, in this analysis stand-

For peer review only - http://bmjopen.bmj.com/site/about/guidelines.xhtml

alone VR is dominant in comparison with usual care (i.e. more effective and less expensive). CEA curves indicate that the probability of cost-effectiveness is 0.72 at a ceiling ratio of 20,000 €/QALY (Supplementary Figure 4), 0.95 at ceiling ratios of 350 €/point of improvement in VSS-SF (Supplementary Figure 5), and 0.95 at ceiling ratios of 4600 €/additional responder based on the VSS-SF (Supplementary Figure 6).

For peer review only

For peer review only - http://bmjopen.bmj.com/site/about/guidelines.xhtml

DISCUSSION

Principal findings

Stand-alone and blended VR resulted in a statistically non-significant gain in QALYs and significantly reduced vestibular symptoms compared to usual care. Total societal costs of both stand-alone and blended VR were statistically non-significantly higher than the costs of usual care. Since stand-alone VR was more effective and less expensive than blended VR, the probability for stand-alone VR to be cost-effective in comparison with usual care was higher. In a sensitivity analysis, where we excluded costs that were unlikely to be related to vestibular symptoms or the use of internet-based VR, stand-alone VR became dominant (i.e. more effective and less expensive) over usual care.

Comparison with existing literature

This is the first study to investigate the cost-effectiveness of internet-based VR for patients with a chronic vestibular syndrome in general practice. This internet-based VR intervention is based on the content of a VR booklet that was previously shown to be highly cost-effective compared to usual care in a randomised controlled trial.[18] Even though both internet-based and booklet-based VR interventions were inexpensive, the total costs of participants in our stand-alone VR and blended VR groups were much higher than in the study evaluating the VR booklet. This difference in costs between the trials probably occurred due to a more narrow definition of costs in the booklet VR trial. In the booklet VR trial, costs only comprised intervention costs and resource use of vestibular-related healthcare. A blinded researcher examined the medical records of participants to assess whether contacts were related to vestibular symptoms. In our trial, we analysed societal costs, which included all healthcare costs (regardless of the reason of contact), informal care costs and lost productivity costs. Total societal costs in the internet-based VR groups were considerably higher than in

For peer review only - http://bmjopen.bmj.com/site/about/guidelines.xhtml

the usual care group. The largest contributor to the difference in costs between stand-alone VR and usual care were hospital admission costs. During the trial, 7 stand-alone VR participants, 2 blended VR participants and 5 usual care participants were admitted to the hospital. Each hospitalisation was evaluated by contacting the participant and/or GP, and none of the hospitalisations were judged to be related to vestibular symptoms and/or internet-based VR. Because care in primary care is generally less expensive, this skewness in costly hospitalisations could have strongly affected the results of the cost-effectiveness analysis. Therefore, we excluded secondary care and medication costs that were likely to be unrelated to vestibular symptoms and/or internet-based VR in the third sensitivity analysis. In this analysis, stand-alone VR was dominant over usual care (i.e. more effective and less costly). For blended VR, the largest difference in costs with usual care was not seen in admissions but in loss of productivity. These relatively high absenteeism rates cannot easily be explained, but contact with a physiotherapist might have changed the way participants prioritised daily activities while experiencing vestibular symptoms. Blended VR participants may have deliberately reduced paid and unpaid work to decrease emotional distress, because they learned this can exacerbate their vestibular symptoms.[46]

Strengths and limitations

Strengths of our study are the pragmatic design of our randomised controlled trial, the societal perspective on costs, the use of multiple outcome measures (QALYs, VSS-SF, clinically relevant response) and conduct of three sensitivity analyses in addition to the main analysis to assess the robustness of the results.

There are also several limitations. First, the trial was powered for the primary outcome measure VSS-SF and not for costs or QALYs. This is reflected in a substantial uncertainty surrounding the relatively large cost differences between groups that did not reach statistical

For peer review only

significance. Secondly, the effectiveness and costs were measured over six months. Based on this study, we cannot ascertain the long-term cost-effectiveness of stand-alone and blended VR versus usual care. Nevertheless, an increase in cost-effectiveness might occur in the long term. Previous VR trials have also shown effectiveness at twelve months,[14, 18] and costs for internet-based VR are not expected to increase during a longer follow-up period. Thirdly, the differences measured in QALYs between the intervention groups and usual care were not significant, unlike the differences in VSS-SF scores. The EQ-5D-5L, a generic utility measure, may not have been sensitive enough to capture changes in quality of life in our population. Certain domains that are important for patients with chronic vestibular syndromes, such as the disease's unpredictability and its impact on social and role functioning, are not measured by the EQ-5D-5L.[47, 48] We also used the Dizziness Handicap inventory[49, 50] in our trial, an outcome measure that specifically quantifies the impact on daily life by vestibular symptoms. We did find significant differences in Dizziness Handicap Inventory scores favouring stand-alone VR and blended VR over usual care (adjusted mean difference at 6 months: -4.9 points; 95% CI -8.4 to -1.3, and -4.5 points; 95% CI -8.0 to -0.9).[25] Using the EQ-5D-5L might have caused us to underestimate the impact of stand-alone and blended VR on quality of life.

Conclusions and implications for research and/or practice

Stand-alone and blended internet-based VR both led to a statistically non-significant gain in QALYs and a significant reduction of vestibular symptoms, but the costs were (non-significantly) higher compared to usual care. After excluding costs unlikely to be related to vestibular symptoms or internet-based VR, stand-alone VR became dominant over usual care in the cost-effectiveness analysis (i.e. more effective and less costly). Internet-based VR is an easily accessible, effective form of treatment that could potentially improve care for a largely

For peer review only - http://bmjopen.bmj.com/site/about/guidelines.xhtml

undertreated group of patients with a chronic vestibular syndrome in general practice. Based on the results of this economic evaluation, stand-alone VR should be preferred over blended VR.

For peer review only

For peer review only - http://bmjopen.bmj.com/site/about/guidelines.xhtml

ADDITIONAL INFORMATION

Competing interests

All authors have completed the Unified Competing Interest form (available on request from the corresponding author) and declare: support from The Netherlands Organisation for Health Research and Development (ZonMw); no financial relationships with any organisations that might have an interest in the submitted work in the previous three years, no other relationships or activities that could appear to have influenced the submitted work.

Funding

This study was funded by The Netherlands Organisation for Health Research and Development (ZonMw) (programme Quality in health care; grant number 839110015). The sponsors did not participate in the study design, data collection, analysis, interpretation, or the preparation or submission of this report.

Ethical approval

The study protocol was approved by the Medical Ethics Committee of the VU University Medical Center. All participants included in the study provided written informed consent.

Copyright

The Corresponding Author has the right to grant on behalf of all authors and does grant on behalf of all authors, an exclusive licence (or non exclusive for government employees) on a worldwide basis to the BMJ Publishing Group Ltd to permit this article (if accepted) to be published in BMJ editions and any other BMJPGL products and sublicences such use and exploit all subsidiary rights, as set out in our licence.

For peer review only - http://bmjopen.bmj.com/site/about/guidelines.xhtml

Transparency declaration

Vincent van Vugt affirms that this manuscript is an honest, accurate, and transparent account of the study being reported; that no important aspects of the study have been omitted; and that any discrepancies from the study as planned (and, if relevant, registered) have been explained.

Contributors

Contributors: ORM obtained the funding and coordinated the study. ORM, VAvV, JCvdW, HEvdH and JEB were involved in the design of the study. VAvV collected the data. JEB and APF analysed the data. VAvV and JEB wrote the first substantial draft of the article and VAvV is the guarantor. ORM, JCvdW, JEB, APF and HEvdH critically revised the manuscript. All authors read and approved the final manuscript.

Data sharing

De-identified individual participant data and data analysis plan available from the corresponding author on reasonable request.

For peer review only

TABLES

Table 1 Pooled mean effect and cost outcomes (SE) stratified for treatment group and differences in mean effect and cost outcomes (95% CI) for the intervention groups compared to usual care.

**Outcome**	**Stand-alone VR N=98**	**Blended VR N=104**	**Usual care N=120**	**Difference stand-alone VR versus usual care**	**Difference blended VR versus usual care**
Effects					
QALY	0.43 (0.008)	0.41 (0.008)	0.41 (0.008)	0.02 (-0.001 ; 0.04)	0.01 (-0.01 ; 0.03)
VSS-SF	8.1 (0.91)	8.5 (0.70)	11.4 (0.95)	3.8 (1.7 ; 6.0)*	3.3 (1.3 ; 5.2)*
Response	0.72 (0.05)	0.61 (0.05)	0.45 (0.95)	0.27 (0.13 ; 0.40)	0.16 (0.02 ; 0.30)
Costs					
Intervention	39 (0)	155 (5)	0 (0)	39	155
Total healthcare	1379 (377)	1116 (229)	901 (162)	478 (-100 ; 1514)	215 (-272 ; 836)
Primary care	304 (42)	362 (59)	315 (37)	-11 (-112 ; 97)	47 (-72 ; 200)
Complementary medicine	22 (7)	8 (3)	29 (11)	-7 (-37 ; 12)	-21 (-54 ; -6)
Outpatient care	104 (19)	147 (78)	116 (20)	-12 (-62 ; 37)	31 (-62 ; 344)
Admissions	682 (350)	22 (19)	129 (95)	553 (102 ; 1563)	-107 (-471 ; 2)
Medication	209 (78)	352 (172)	171 (57)	38 (-105 ; 288)	181 (-46 ; 817)
Home care	58 (22)	224 (107)	140 (43)	-82 (-193 ; -5)	84 (-59 ; 460)
Informal care	88 (30)	187 (93)	112 (38)	-24 (-123 ; 56)	75 (-53 ; 392)
Total lost productivity	2061 (538)	2521 (541)	2049 (443)	12 (-1260 ; 1439)	472 (-846 ; 1825)
Absenteeism	241 (133)	931 (331)	486 (199)	-245 (-776 ; 190)	445 (-186 ; 1337)
Presenteeism	10 (3)	14 (5)	15 (5)	-5 (-19 ; 4)	-1 (-15 ; 12)
Unpaid work	1810 (518)	1576 (229)	1548 (361)	262 (-807 ; 1618)	28 (-981 ; 1069)
Total societal	3567 (701)	3979 (667)	3063 (520)	504 (-1082 ; 2268)	916 (-663 ; 2596)

SE = standard error; CI = confidence interval; VR = vestibular rehabilitation; VSS-SF = Vertigo Symptom Scale – Short Form, range 0-60, clinically relevant difference 3 points; Response = percentage of participants with a decrease of ≥3 points in VSS-SF between baseline and six months

* Due to a different method of analysis VSS-SF scores slightly differ from the previously reported clinical effectiveness analysis. [25]

For peer review only - http://bmjopen.bmj.com/site/about/guidelines.xhtml

Table 2 Cost-effectiveness outcomes for stand-alone VR compared to usual care.

Outcome	ΔC (95% CI)	ΔE (95% CI)	ICER	CE plane			
				NE	SE	SW	NW
**Main analysis – societal perspective**							
QALY	504 (-1064 ; 2303)	0.02 (-0.001 ; 0.04)	24,161	69%	28%	0%	3%
VSS-SF	504 (-1052 ; 2294)	3.8 (1.7 ; 6.0)	132	72%	28%	0%	0%
Response*	504 (-1057 ; 2282)	0.27 (0.13 ; 0.40)	1895	72%	28%	0%	0%
**SA1 – healthcare perspective**							
QALY	478 (-103 ; 1510)	0.02 (-0.001 ; 0.04)	22,936	86%	11%	0%	3%
VSS-SF	478 (-94 ; 1527)	3.8 (1.7 ; 6.0)	126	89%	11%	0%	0%
Response*	478 (-104 ; 1520)	0.27 (0.13 ; 0.40)	1799	89%	11%	0%	0%
**SA2 – societal perspective, outliers recoded as missings**							
QALY	241 (-1194 ; 1838)	0.02 (-0.001 ; 0.04)	11,388	58%	39%	0%	3%
VSS-SF	241 (-1184 ; 1851)	3.7 (1.6 ; 5.7)	66	60%	40%	0%	0%
Response*	241 (-1195 ; 1832)	0.27 (0.13 ; 0.40)	910	61%	39%	0%	0%
**SA3 – societal perspective, only costs related to vestibular symptoms**							
QALY	-75 (-1425 ; 1450)	0.02 (-0.001 ; 0.04)	-3603	43%	54%	1%	2%
VSS-SF	-75 (-1430 ; 1463)	3.8 (1.7 ; 6.0)	-20	45%	55%	0%	0%
Response*	-75 (-1432 ; 1453)	0.27 (0.13 ; 0.40)	-283	45%	55%	0%	0%

* Response was defined as ≥3 points improvement on the Vertigo Symptom Scale – Short Form after six months
ΔC = cost difference between stand-alone VR and usual care; ΔE = effect difference between stand-alone VR and usual care; CI = confidence interval; ICER = incremental cost-effectiveness ratio; CE = cost-effectiveness; NE = northeast (more expensive & more effective); SA1 = sensitivity analysis with only healthcare costs included; SA2 = sensitivity analysis with extreme outliers recoded as missings; SA3 = sensitivity analysis with secondary care and medication costs excluded; SE = southeast (less expensive & more effective); SW = southwest (less expensive & less effective); NW = northwest (more expensive & less effective); VR = vestibular rehabilitation; VSS-SF = Vertigo Symptom Scale – Short Form; QALY = quality-adjusted life-years

For peer review only - http://bmjopen.bmj.com/site/about/guidelines.xhtml

Table 3 Cost-effectiveness outcomes for blended VR compared to usual care.

**Outcome**	**ΔC (95% CI)**	**ΔE (95% CI)**	**ICER**	**CE plane**			
				**NE**	**SE**	**SW**	**NW**
**Main analysis – societal perspective**							
QALY	916 (-660 ; 2579)	0.007 (-0.01 ; 0.03)	123,335	62%	12%	1%	25%
VSS-SF	916 (-655 ; 2610)	3.3 (1.3 ; 5.2)	280	86%	14%	0%	0%
Response*	916 (-658 ; 2583)	0.16 (0.02 ; 0.30)	5599	85%	13%	0%	0%
**SA1 – healthcare perspective**							
QALY	215 (-273 ; 820)	0.007 (-0.01 ; 0.03)	28,848	55%	19%	3%	23%
VSS-SF	215 (-263 ; 828)	3.3 (1.4 ; 5.2)	65	78%	22%	0%	0%
Response*	215 (-263 ; 832)	0.16 (0.02 ; 0.30)	1310	77%	22%	0%	1%
**SA2 – societal perspective, outliers recoded as missings**							
QALY	1140 (-404 ; 2811)	0.007 (-0.01 ; 0.03)	156,954	66%	8%	1%	25%
VSS-SF	1140 (-404 ; 2811)	3.1 (1.3 ; 5.0)	366	91%	9%	0%	0%
Response*	1140 (-401 ; 2789)	0.16 (0.02 ; 0.30)	6988	91%	8%	0%	1%
**SA3 – societal perspective, only costs related to vestibular symptoms**							
QALY	810 (-653 ; 2400)	0.007 (-0.01 ; 0.03)	109,121	61%	13%	1%	24%
VSS-SF	810 (-653 ; 2406)	3.3 (1.4 ; 5.2)	248	85%	15%	0%	0%
Response*	810 (-650 ; 2410)	0.16 (0.02 ; 0.30)	4954	84%	15%	0%	1%

* Response was defined as ≥3 points improvement on the Vertigo Symptom Scale – Short Form after six months
ΔC = cost difference between stand-alone VR and usual care; ΔE = effect difference between stand-alone VR and usual care; CI = confidence interval; ICER = incremental cost-effectiveness ratio; CE = cost-effectiveness; NE = northeast (more expensive & more effective); SA1 = sensitivity analysis with only healthcare costs included; SA2 = sensitivity analysis with extreme outliers recoded as missings; SA3 = sensitivity analysis with secondary care and medication costs excluded; SE = southeast (less expensive & more effective); SW = southwest (less expensive & less effective); NW = northwest (more expensive & less effective); VR = vestibular rehabilitation; VSS-SF = Vertigo Symptom Scale – Short Form; QALY = quality-adjusted life-years

For peer review only

For peer review only - http://bmjopen.bmj.com/site/about/guidelines.xhtml

Figure legends

Figure 1. Main analysis – societal perspective. Cost-effectiveness acceptability curve for the Vertigo Symptom Scale - Short Form comparing stand-alone VR with usual care.

VR = vestibular rehabilitation

Figure 2. Main analysis – societal perspective. Cost-effectiveness acceptability curve for the Vertigo Symptom Scale - Short Form comparing blended VR with usual care.

VR = vestibular rehabilitation

For peer review only

For peer review only - http://bmjopen.bmj.com/site/about/guidelines.xhtml

REFERENCES

1. Neuhauser HK. The epidemiology of dizziness and vertigo. Handb Clin Neurol. 2016;137:67-82.
2. Hanley K, O' Dowd T. Symptoms of vertigo in general practice: a prospective study of diagnosis. Br J Gen Pract. 2002;52(483):809-12.
3. Bisdorff A, Von Brevern M, Lempert T, et al. Classification of vestibular symptoms: towards an international classification of vestibular disorders. J Vestib Res. 2009;19(1-2):1-13.
4. Saber Tehrani AS, Coughlan D, Hsieh YH, et al. Rising annual costs of dizziness presentations to U.S. emergency departments. Acad Emerg Med. 2013;20(7):689-96.
5. Wang X, Strobl R, Holle R, et al. Vertigo and dizziness cause considerable more health care resource use and costs: results from the KORA FF4 study. J Neurol. 2019.
6. Bisdorff A. Vestibular symptoms and history taking. Handb Clin Neurol. 2016;137:83-90.
7. Bisdorff AR, Staab JP, Newman-Toker DE. Overview of the International Classification of Vestibular Disorders. Neurol Clin. 2015;33(3):541-50, vii.
8. Edlow JA, Gurley KL, Newman-Toker DE. A New Diagnostic Approach to the Adult Patient with Acute Dizziness. J Emerg Med. 2018;54(4):469-83.
9. Bhattacharyya N, Gubbels SP, Schwartz SR, et al. Clinical Practice Guideline: Benign Paroxysmal Positional Vertigo (Update). Otolaryngol Head Neck Surg. 2017;156(3_suppl):S1-S47.
10. Hall CD, Herdman SJ, Whitney SL, et al. Vestibular rehabilitation for peripheral vestibular hypofunction: an evidence-based clinical practice guideline: from the American Physical Therapy Association Neurology section. J Neurol Phys Ther. 2016;40(2):124-55.
11. National Institute for Health and Care Excellence (NICE) Vestibular neuronitis. Nice, 2011. [https://cks.nice.org.uk/vestibular-neuronitis]
12. Summary of the Dutch College of General Practitioners' practice guideline 'Dizziness' (in Dutch). Utrecht: Dutch college of general practitioners, 2017. [https://www.nhg.org/system/files/content/nhg_org/uploads/standaard/pdf/m75_2.pdf 2017]
13. van Vugt VA, van der Horst HE, Payne RA, Maarsingh OR. Chronic vertigo: treat with exercise, not drugs. BMJ. 2017;358:j3727.
14. McDonnell MN, Hillier SL. Vestibular rehabilitation for unilateral peripheral vestibular dysfunction. Cochrane Database Syst Rev. 2015;1:CD005397.
15. Porciuncula F, Johnson CC, Glickman LB. The effect of vestibular rehabilitation on adults with bilateral vestibular hypofunction: a systematic review. J Vestib Res. 2012;22(5-6):283-98.
16. van Vugt VA, Diaz Nerio PM, van der Wouden JC, et al. Use of canalith repositioning manoeuvres and vestibular rehabilitation: a GP survey. Scand J Prim Health Care. 2017;35(1):19-26.
17. Jayarajan V, Rajenderkumar D. A survey of dizziness management in General Practice. J Laryngol Otol. 2003;117(8):599-604.
18. Yardley L, Barker F, Muller I, et al. Clinical and cost effectiveness of booklet based vestibular rehabilitation for chronic dizziness in primary care: single blind, parallel group, pragmatic, randomised controlled trial. BMJ. 2012;344:e2237.
19. Geraghty AW, Kirby S, Essery R, et al. Internet-based vestibular rehabilitation for adults aged 50 years and over: a protocol for a randomised controlled trial. BMJ Open. 2014;4(7):e005871.

For peer review only - http://bmjopen.bmj.com/site/about/guidelines.xhtml

20. Geraghty AWA, Essery R, Kirby S, et al. Internet-based vestibular rehabilitation for older Adults with chronic dizziness: a randomized controlled trial in primary care. Ann Fam Med. 2017;15(3):209-16.
21. van der Vaart R, Witting M, Riper H, et al. Blending online therapy into regular face-to-face therapy for depression: content, ratio and preconditions according to patients and therapists using a Delphi study. BMC Psychiatry. 2014;14:355.
22. van Ballegooijen W, Cuijpers P, van Straten A, et al. Adherence to Internet-based and face-to-face cognitive behavioural therapy for depression: a meta-analysis. PLoS One. 2014;9(7):e100674.
23. Wilhelmsen M, Lillevoll K, Risor MB, et al. Motivation to persist with internet-based cognitive behavioural treatment using blended care: a qualitative study. BMC Psychiatry. 2013;13:296.
24. van Vugt VA, van der Wouden JC, Bosmans JE, et al. Guided and unguided internet-based vestibular rehabilitation versus usual care for dizzy adults of 50 years and older: a protocol for a three-armed randomised trial. BMJ Open. 2017;7(1):e015479.
25. van Vugt VA, van der Wouden JC, Essery R, et al. Internet based vestibular rehabilitation with and without physiotherapy support for adults aged 50 and older with a chronic vestibular syndrome in general practice: three armed randomised controlled trial. BMJ. 2019;367:l5922.
26. Husereau D, Drummond M, Petrou S, et al. Consolidated Health Economic Evaluation Reporting Standards (CHEERS) statement. BMJ. 2013;346:f1049.
27. Richardson J. Multiattribute utility instruments and their use. Encyclopedia of Health Economics San Diego: Elsevier Science; 2014.
28. Herdman M, Gudex C, Lloyd A, et al. Development and preliminary testing of the new five-level version of EQ-5D (EQ-5D-5L). Qual Life Res. 2011;20(10):1727-36.
29. Versteegh M, Vermeulen K, Evers S, et al. Dutch tariff for the five-level version of EQ-5D. Value Health. 2016;19(4):343-52.
30. Yardley L, Masson E, Verschuur C, et al. Symptoms, anxiety and handicap in dizzy patients: development of the vertigo symptom scale. J Psychosom Res. 1992;36(8):731-41.
31. Wilhelmsen K, Strand LI, Nordahl SH, et al. Psychometric properties of the Vertigo symptom scale - Short form. BMC Ear Nose Throat Disord. 2008;8:2.
32. Yardley L, Donovan-Hall M, Smith HE, et al. Effectiveness of primary care-based vestibular rehabilitation for chronic dizziness. Ann Intern Med. 2004;141(8):598-605.
33. Yardley L, Kirby S. Evaluation of booklet-based self-management of symptoms in Meniere disease: a randomized controlled trial. Psychosom Med. 2006;68(5):762-9.
34. Bouwmans C, Hakkaart-van Roijen L, Koopmanschap M, et al. Handleiding *i*MTA Medical Cost Questionnaire (*i*MCQ). 2013.
35. Bouwmans C, Krol M, Severens H, et al. The iMTA Productivity Cost Questionnaire: A Standardized Instrument for Measuring and Valuing Health-Related Productivity Losses. Value Health. 2015;18(6):753-8.
36. Aronsson G, Gustafsson K, Dallner M. Sick but yet at work. An empirical study of sickness presenteeism. J Epidemiol Community Health. 2000;54(7):502-9.
37. Hakkaart-van Roijen L, Tan S, Bouwmans C. Handleiding voor kostenonderzoek [Dutch manual for costing in economic evaluations]. . 2011.
38. Medicijnkosten Nederland [Internet]. Medicijnkosten.nl. 2019 [cited 6 November 2019]. Available from: http://www.medicijnkosten.nl/
39. Koopmanschap MA, Rutten FF, van Ineveld BM, et al. The friction cost method for measuring indirect costs of disease. J Health Econ. 1995;14(2):171-89.
40. White IR, Royston P, Wood AM. Multiple imputation using chained equations: Issues and guidance for practice. Stat Med. 2011;30(4):377-99.

For peer review only

For peer review only - http://bmjopen.bmj.com/site/about/guidelines.xhtml

41. Madley-Dowd P, Hughes R, Tilling K, et al. The proportion of missing data should not be used to guide decisions on multiple imputation. J Clin Epidemiol. 2019;110:63-73.
42. Rubin D. Multiple imputation for nonresponse in surveys. Hoboken, N.J.: John Wiley; 2011
43. Black WC. The CE plane: a graphic representation of cost-effectiveness. Med Decis Making. 1990;10(3):212-4.
44. Fenwick E, O'Brien BJ, Briggs A. Cost-effectiveness acceptability curves--facts, fallacies and frequently asked questions. Health Econ. 2004;13(5):405-15.
45. Cost-effectiveness in practice: National Health Care Institute of The Netherlands; 2015 [Available from: https://english.zorginstituutnederland.nl/publications/reports/2015/06/16/cost-effectiveness-in-practice.]
46. Meli A, Zimatore G, Badaracco C, et al. Effects of vestibular rehabilitation therapy on emotional aspects in chronic vestibular patients. J Psychosom Res. 2007;63(2):185-90.
47. Ciorba A, Bianchini C, Scanelli G, et al. The impact of dizziness on quality-of-life in the elderly. Eur Arch Otorhinolaryngol. 2017;274(3):1245-50.
48. Finch AP, Brazier JE, Mukuria C, et al. An Exploratory Study on Using Principal-Component Analysis and Confirmatory Factor Analysis to Identify Bolt-On Dimensions: The EQ-5D Case Study. Value Health. 2017;20(10):1362-75.
49. Jacobson GP, Newman CW. The development of the Dizziness Handicap Inventory. Arch Otolaryngol Head Neck Surg. 1990;116(4):424-7.
50. Tamber AL, Wilhelmsen KT, Strand LI. Measurement properties of the Dizziness Handicap Inventory by cross-sectional and longitudinal designs. Health Qual Life Outcomes. 2009;7:101.

For peer review only

For peer review only - http://bmjopen.bmj.com/site/about/guidelines.xhtml

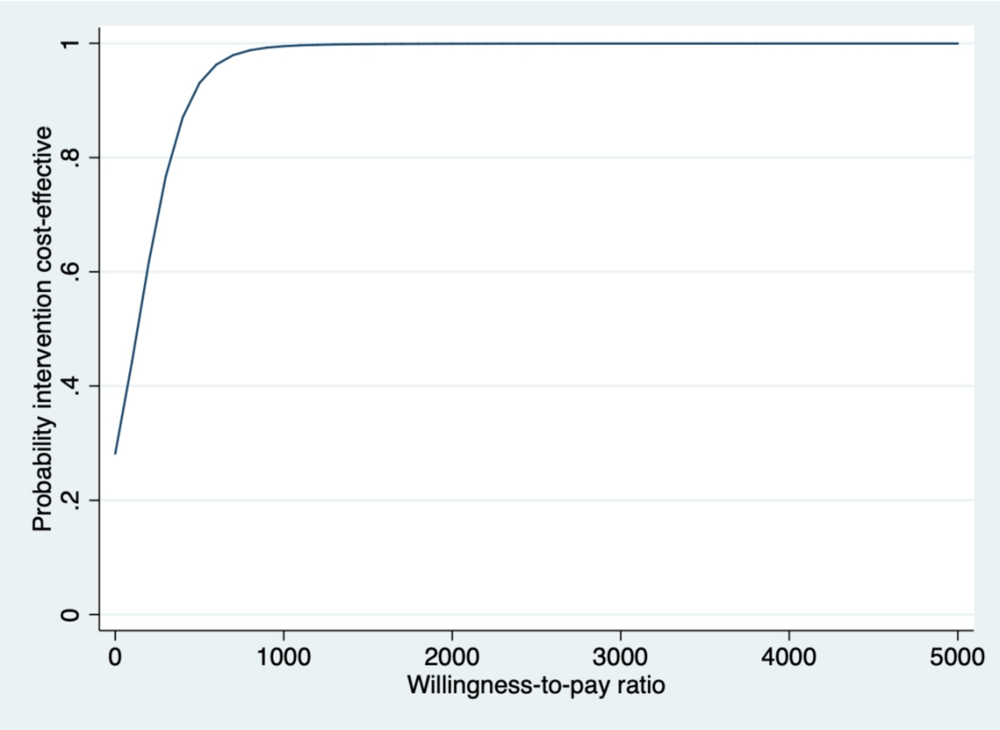

Figure 1. Main analysis – societal perspective. Cost-effectiveness acceptability curve for the Vertigo Symptom Scale - Short Form comparing stand-alone VR with usual care.
VR = vestibular rehabilitation

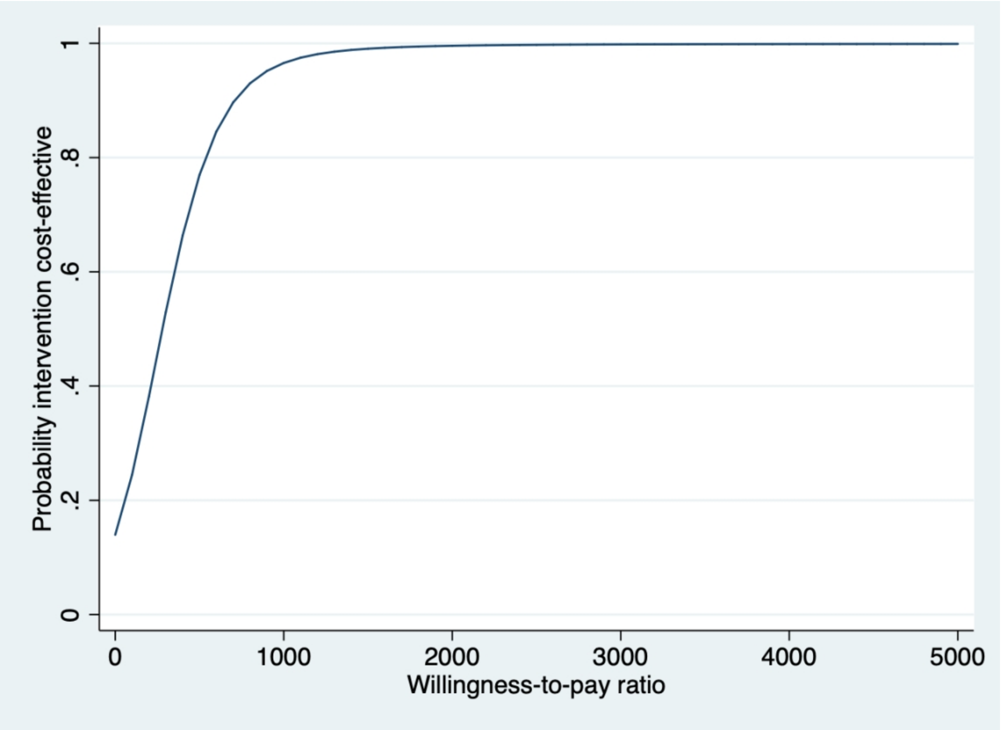

Figure 2. Main analysis – societal perspective. Cost-effectiveness acceptability curve for the Vertigo Symptom Scale - Short Form comparing blended VR with usual care.
VR = vestibular rehabilitation

Supplementary Figure 1. Overview of enrolment, allocation and follow-up.

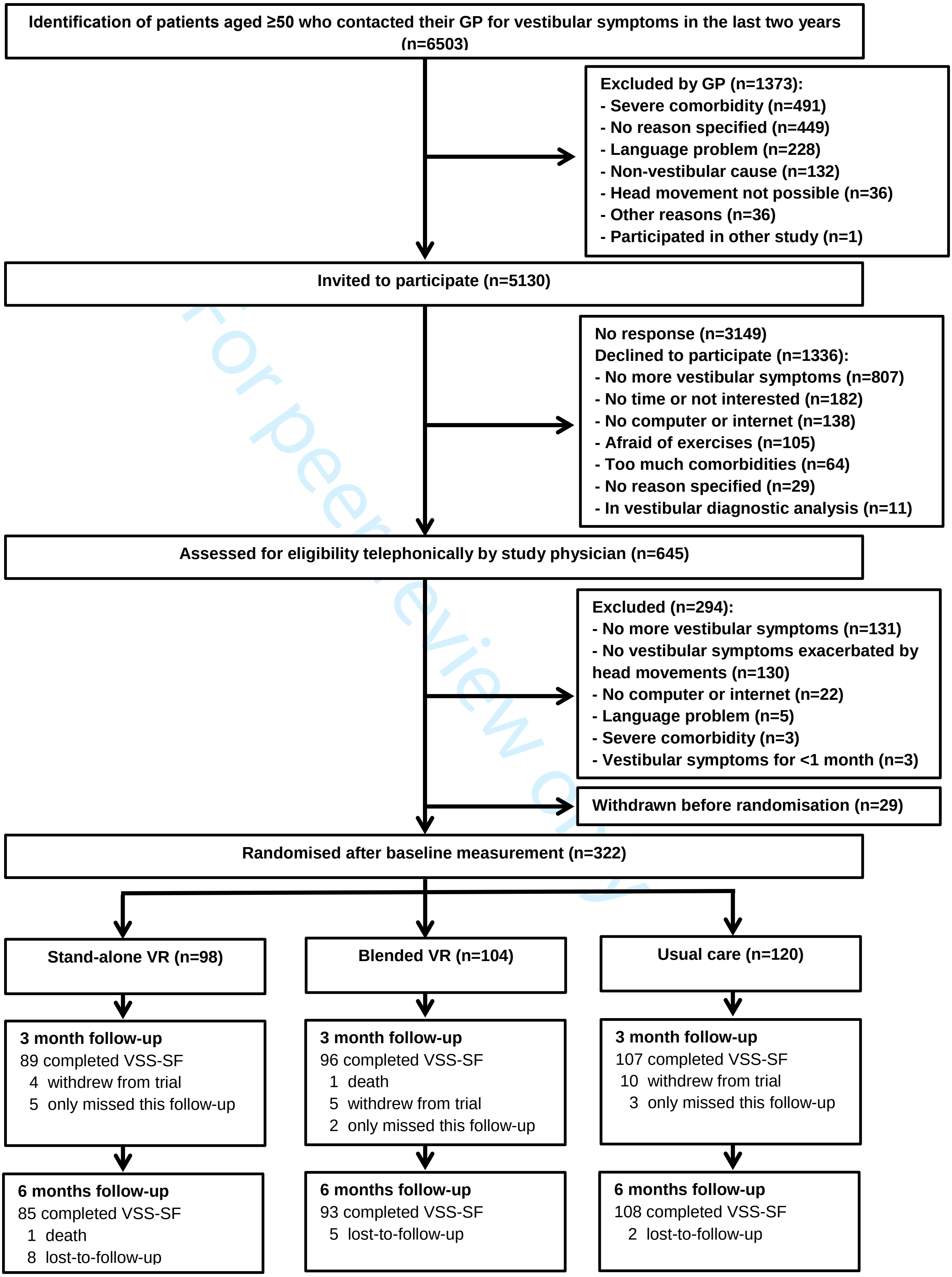

VR = vestibular rehabilitation; VSS-SF = Vertigo Symptom Scale Short Form

For peer review only - http://bmjopen.bmj.com/site/about/guidelines.xhtml

Supplementary Figure 2. Main analysis – societal perspective. Cost-effectiveness acceptability curve for clinically relevant response (improvement of ≥ 3 points on Vertigo Symptom Scale - Short Form) comparing stand-alone vestibular rehabilitation with usual care.

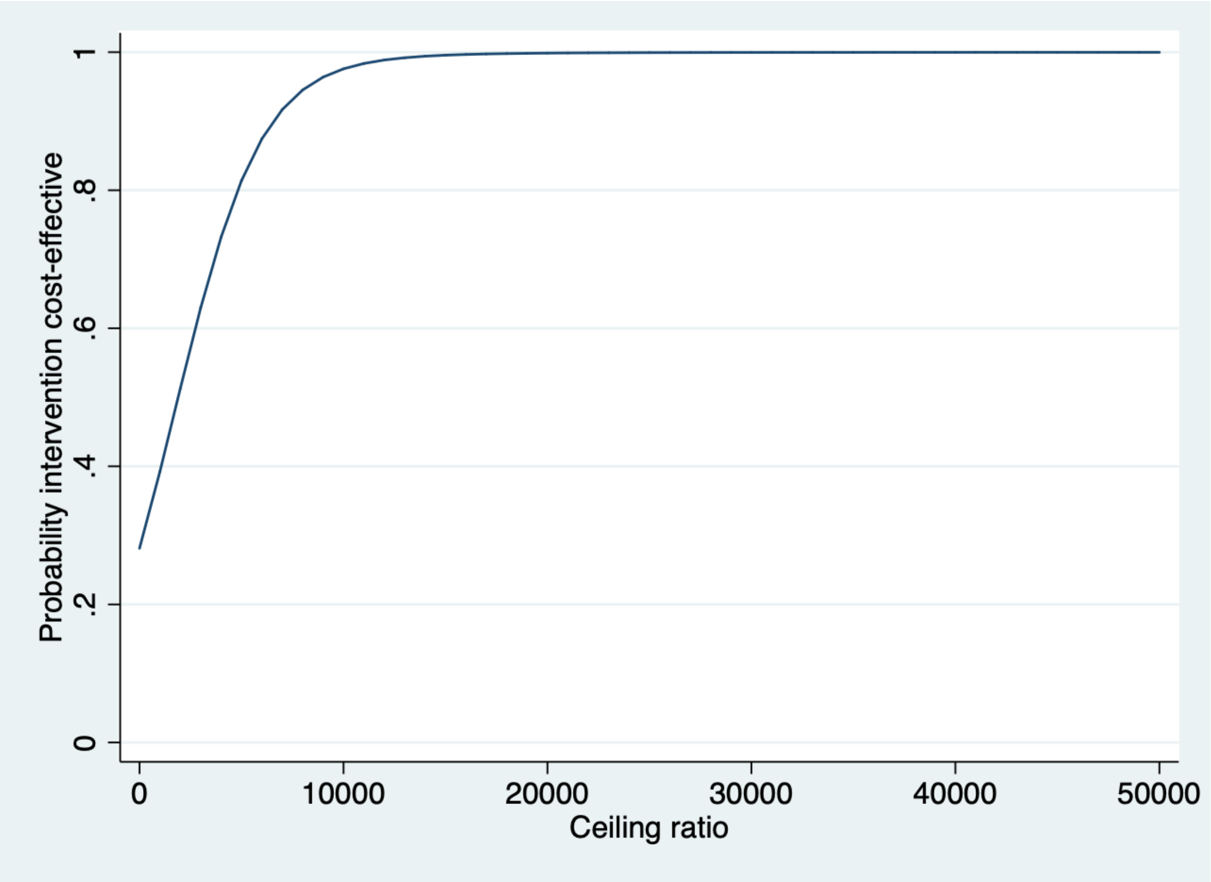

Supplementary Figure 3. Main analysis – societal perspective. Cost-effectiveness acceptability curve for clinically relevant response (improvement of ≥ 3 points on Vertigo Symptom Scale - Short Form) comparing blended vestibular rehabilitation with usual care.

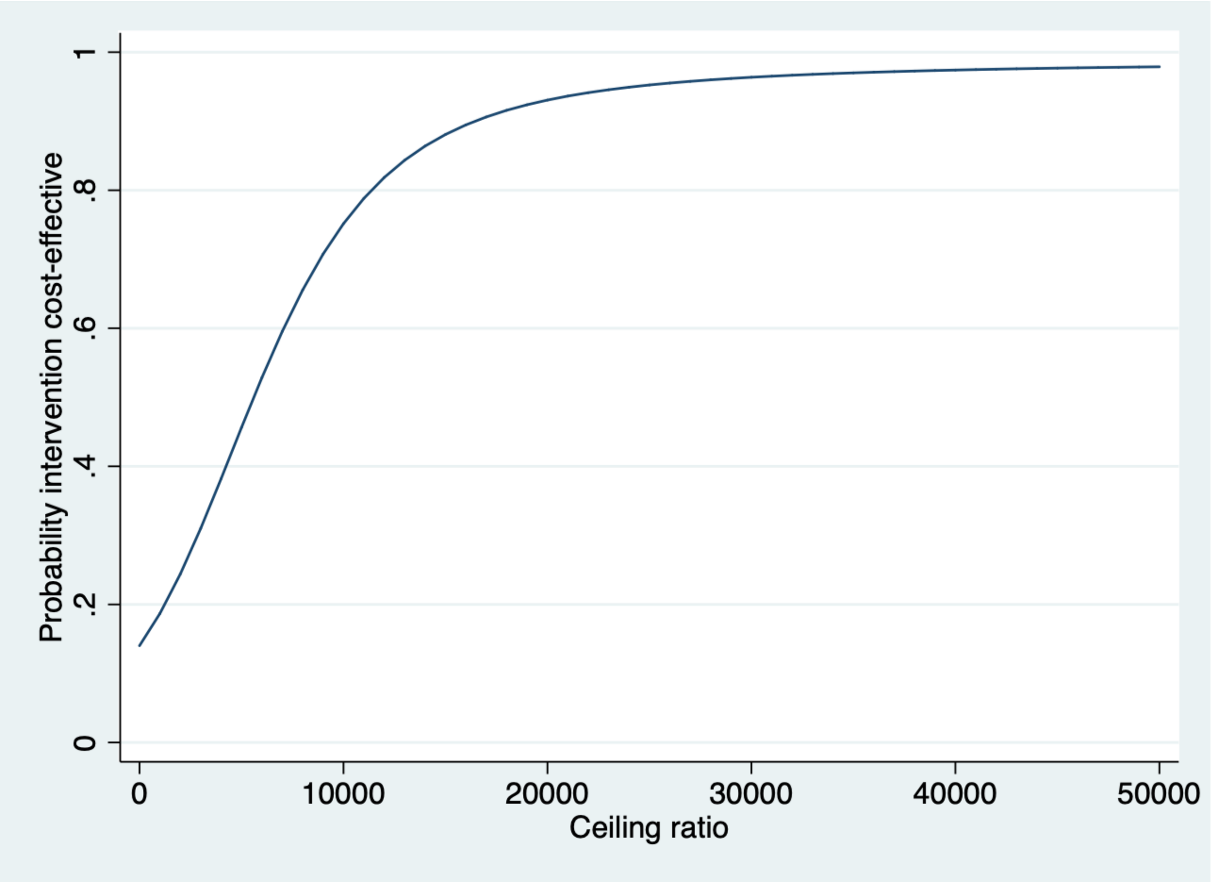

Supplementary Figure 4. SA3 – societal perspective. Cost-effectiveness acceptability curve for Quality-Adjusted Life Years comparing stand-alone vestibular rehabilitation with usual care.

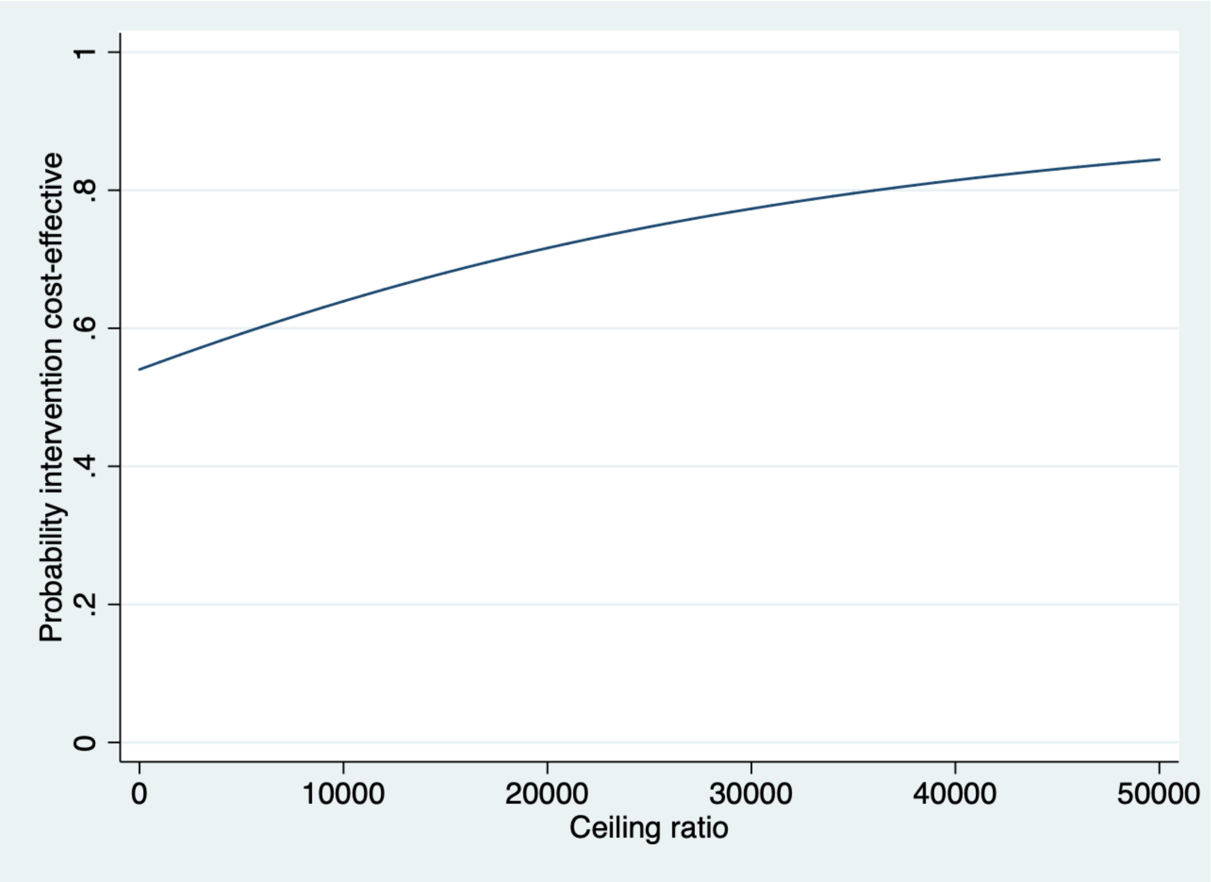

SA3 = sensitivity analysis with secondary care and medication costs excluded

For peer review only

Supplementary Figure 5. SA3 – societal perspective. Cost-effectiveness acceptability curve for the Vertigo Symptom Scale - Short Form comparing stand-alone vestibular rehabilitation with usual care.

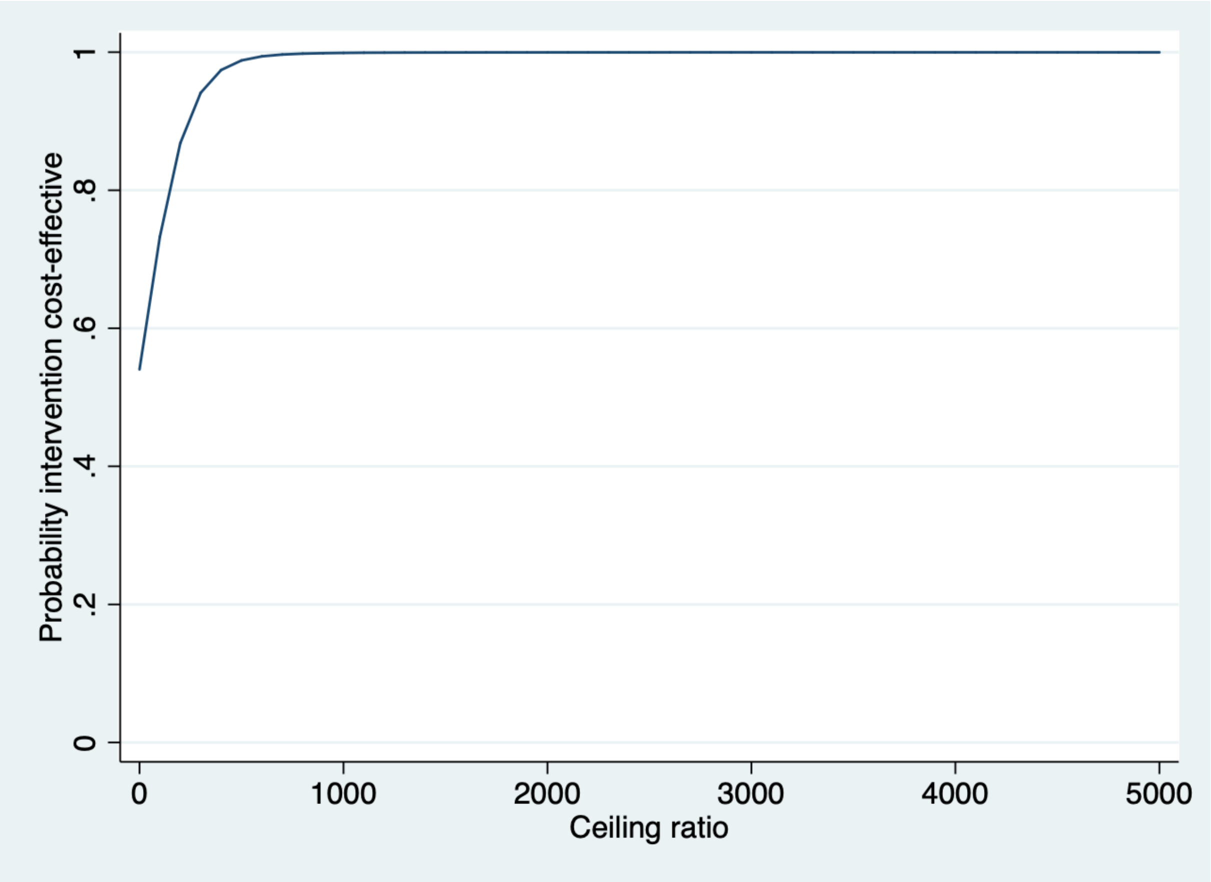

SA3 = sensitivity analysis with secondary care and medication costs excluded

For peer review only

Supplementary Figure 6. SA3 – societal perspective. Cost-effectiveness acceptability curve for clinically relevant response (improvement of ≥ 3 points on Vertigo Symptom Scale - Short Form) comparing stand-alone vestibular rehabilitation with usual care.

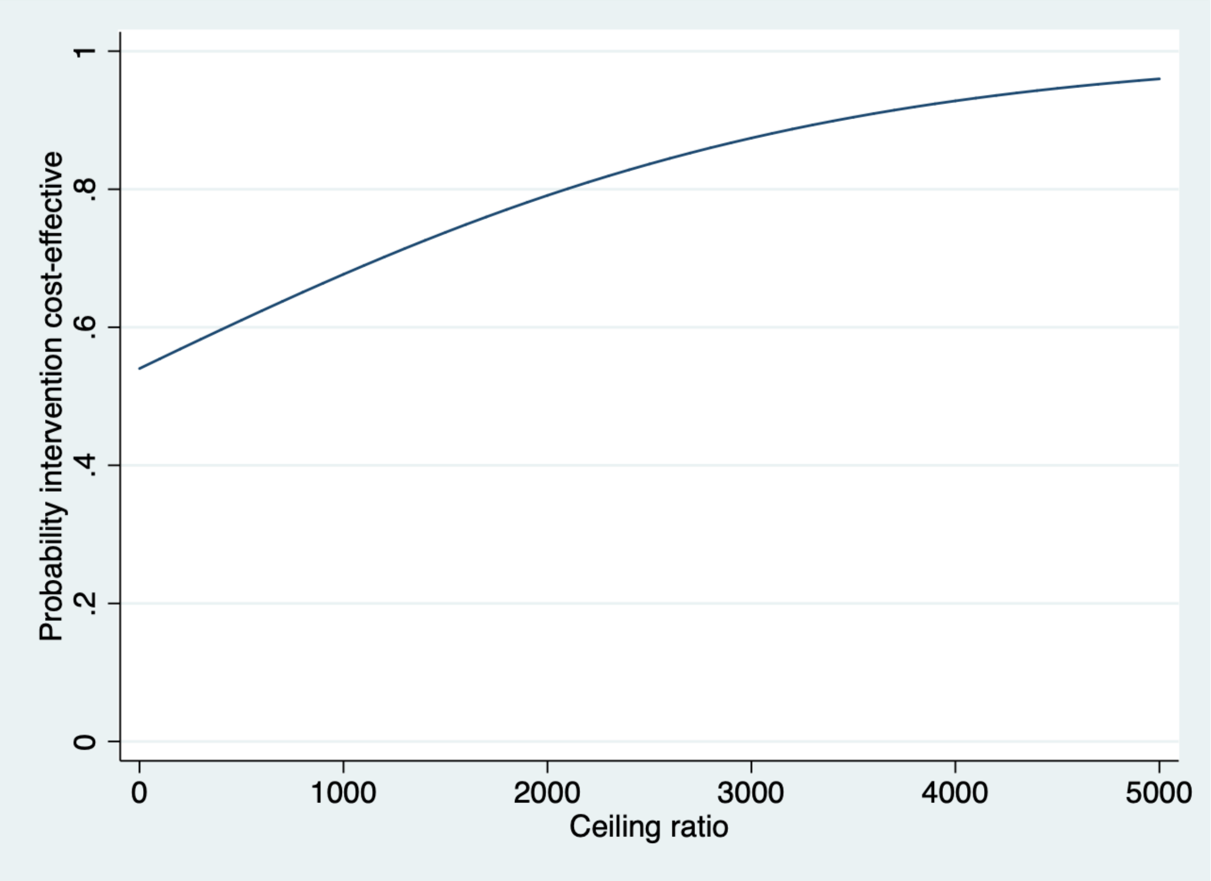

SA3 = sensitivity analysis with secondary care and medication costs excluded

For peer review only

Supplementary Table 1 Baseline characteristics. Figures are numbers (percentages) unless stated otherwise.

		Stand-alone VR (N=98)	Blended VR (N=104)	Usual care (N=120)	Total sample (N=322)
Mean (SD) age (years)		66.7 (9.5)	67.4 (9.8)	67.0 (9.4)	67.0 (9.5)
Female		64 (65)	69 (66)	64 (53)	197 (61)
Level of education					
	Low	33 (34)	37 (36)	36 (30)	106 (33)
	Middle	25 (26)	31 (30)	30 (25)	86 (27)
	High	40 (41)	36 (35)	54 (45)	130 (40)
Living situation					
	Alone	34 (35)	33 (32)	35 (29)	102 (32)
	With partner	64 (65)	71 (68)	85 (71)	220 (68)
Number of chronic diseases*					
	0	59 (60)	64 (62)	63 (53)	186 (58)
	1	28 (29)	32 (31)	41 (34)	101 (31)
	2	8 (8)	4 (4)	12 (10)	24 (7)
	≥3	3 (3)	4 (4)	4 (3)	11 (3)
Time since vestibular diagnosis**					
	One to six months	15 (15)	22 (21)	13 (11)	50 (16)
	Six months to two years	28 (29)	27 (26)	39 (33)	94 (29)
	Two years to 10 years	31 (32)	44 (42)	48 (40)	123 (38)
	More than 10 years	23 (24)	11 (11)	18 (15)	52 (16)
Self-reported vestibular diagnosis**					
	No known diagnosis	67 (68)	69 (66)	77 (64)	213 (66)
	Benign paroxysmal positional vertigo	11 (11)	17 (16)	22 (18)	50 (16)
	Meniere's disease	9 (9)	9 (9)	10 (8)	28 (9)
	Vestibular neuritis	6 (6)	4 (4)	7 (6)	17 (5)
	PPPD	0 (0)	1 (1)	0 (0)	1 (0)
	Other^	4 (4)	4 (4)	2 (2)	10 (3)
Disorders at baseline according to PHQ		14 (14)	16 (15)	23 (19)	53 (17)
	Panic disorder	2 (2)	3 (3)	5 (4)	10 (3)
	Generalised anxiety disorder	12 (12)	15 (14)	19 (16)	46 (14
	Major depressive disorder	5 (5)	6 (6)	9 (8)	20 (6)

PPPD = persistent postural-perceptual dizziness; PHQ = patient health questionnaire.
*Chronic non-specific lung disease, cardiac disease, peripheral arterial disease, stroke, diabetes mellitus, arthritis, and cancer.
**Data on this variable was missing for three participants: stand-alone VR (n=1), usual care (n=2).
^ Traumatic brain injury, cerebrovascular accident, Parkinson's disease, bacterial meningitis, and vestibular organ surgical procedures.

For peer review only

Supplementary Table 2 Mean costs (SE) stratified for treatment group after recoding outliers as missing (SA2) and cost outcomes (95% CI) for the intervention groups compared to usual care.

Cost category	Stand-alone VR N=98	Blended VR N=104	Usual care N=120	Difference stand-alone VR versus usual care (95% CI)	Difference blended VR versus usual care (95% CI)
Healthcare costs	846 (118)	1428 (232)	775 (103)	71 (-221 ; 368)	653 (-254 ; 1257)
Lost productivity costs	2465 (684)	2992 (766)	2083 (462)	383 (-998 ; 2199)	910 (-505 ; 3026)
Total societal costs	3311 (736)	4420 (825)	2857 (502)	454 (-1019 ; 2399)	1563 (-47 ; 3764)

SE = standard error; VR = vestibular rehabilitation; CI = confidence interval

For peer review only

CHEERS checklist—Items to include when reporting economic evaluations of health interventions

Section/item	Item No	Recommendation	Reported on page No/ line No
**Title and abstract**			
Title	1	Identify the study as an economic evaluation or use more specific terms such as “cost-effectiveness analysis”, and describe the interventions compared.	Page 1, line 1 to 3
Abstract	2	Provide a structured summary of objectives, perspective, setting, methods (including study design and inputs), results (including base case and uncertainty analyses), and conclusions.	Page 2, line 32 to page 3, line 64
**Introduction**			
Background and objectives	3	Provide an explicit statement of the broader context for the study.	Page 4, line 77 to 83
		Present the study question and its relevance for health policy or practice decisions.	Page 5, line 114 to 116
**Methods**			
Target population and subgroups	4	Describe characteristics of the base case population and subgroups analysed, including why they were chosen.	Page 6, line 128 to 137
Setting and location	5	State relevant aspects of the system(s) in which the decision(s) need(s) to be made.	Page 6, line 119 to 130
Study perspective	6	Describe the perspective of the study and relate this to the costs being evaluated.	Page 9, line 203 to 210
Comparators	7	Describe the interventions or strategies being compared and state why they were chosen.	Page 6, line 139 to Page 7, line 161
Time horizon	8	State the time horizon(s) over which costs and consequences are being evaluated and say why appropriate.	Page 9, line 203 to 210
Discount rate	9	Report the choice of discount rate(s) used for costs and outcomes and say why appropriate.	Page 9, line 203 to 210
Choice of health outcomes	10	Describe what outcomes were used as the measure(s) of benefit in the evaluation and their relevance for the type of analysis performed.	Page 7, line 163 to Page 9, line 201
Measurement of effectiveness	11a	*Single study-based estimates:* Describe fully the design features of the single effectiveness study and why the single study was a sufficient source of clinical effectiveness data.	Page 6, line 119 to Page 7, line 161
	11b	*Synthesis-based estimates*: Describe fully the methods used for identification of included studies and synthesis of clinical effectiveness data.	Not applicable
Measurement and valuation of preference based outcomes	12	If applicable, describe the population and methods used to elicit preferences for outcomes.	Not applicable
Estimating resources and costs	13a	*Single study-based economic evaluation:* Describe approaches used to estimate resource use associated with the alternative interventions. Describe primary or secondary research methods for valuing each resource item in terms of its unit cost. Describe any adjustments made to approximate to opportunity costs.	Page 8, line 179 to Page 9, line 201
	13b	*Model-based economic evaluation:* Describe approaches and data sources used to estimate	Not applicable

For peer review only

Section/item	Item No	Recommendation	Reported on page No/ line No
		resource use associated with model health states. Describe primary or secondary research methods for valuing each resource item in terms of its unit cost. Describe any adjustments made to approximate to opportunity costs.	
Currency, price date, and conversion	14	Report the dates of the estimated resource quantities and unit costs. Describe methods for adjusting estimated unit costs to the year of reported costs if necessary. Describe methods for converting costs into a common currency base and the exchange rate.	Page 8, line 179 to Page 9, line 201
Choice of model	15	Describe and give reasons for the specific type of decision-analytical model used. Providing a figure to show model structure is strongly recommended.	Page 9, line 203 to page 10, line 234
Assumptions	16	Describe all structural or other assumptions underpinning the decision-analytical model.	Page 9, line 203 to page 10, line 234
Analytical methods	17	Describe all analytical methods supporting the evaluation. This could include methods for dealing with skewed, missing, or censored data; extrapolation methods; methods for pooling data; approaches to validate or make adjustments (such as half cycle corrections) to a model; and methods for handling population heterogeneity and uncertainty.	Page 9, line 203 to page 10, line 234
**Results**			
Study parameters	18	Report the values, ranges, references, and, if used, probability distributions for all parameters. Report reasons or sources for distributions used to represent uncertainty where appropriate. Providing a table to show the input values is strongly recommended.	Page 12, line 256 to page 15, line 346 Table 1, Table 2 and Table 3
Incremental costs and outcomes	19	For each intervention, report mean values for the main categories of estimated costs and outcomes of interest, as well as mean differences between the comparator groups. If applicable, report incremental cost-effectiveness ratios.	Page 12, line 256 to page 15, line 346 Table 1, Table 2 and Table 3
Characterising uncertainty	20a	*Single study-based economic evaluation:* Describe the effects of sampling uncertainty for the estimated incremental cost and incremental effectiveness parameters, together with the impact of methodological assumptions (such as discount rate, study perspective).	Page 12, line 256 to page 15, line 346 Table 1, Table 2 and Table 3
	20b	*Model-based economic evaluation:* Describe the effects on the results of uncertainty for all input parameters, and uncertainty related to the structure of the model and assumptions.	Not applicable
Characterising heterogeneity	21	If applicable, report differences in costs, outcomes, or cost-effectiveness that can be explained by variations between subgroups of patients with different baseline characteristics or other observed variability in effects that are not reducible by more information.	Not applicable
**Discussion**			
Study findings, limitations, generalisability, and current knowledge	22	Summarise key study findings and describe how they support the conclusions reached. Discuss limitations and the generalisability of the findings and how the findings fit with current knowledge.	Page 16, line 348 to Page 19, line 425
**Other**			
Source of funding	23	Describe how the study was funded and the role of	Page 20, line 436 to 440

For peer review only

Section/item	Item No	Recommendation	Reported on page No/ line No
		the funder in the identification, design, conduct, and reporting of the analysis. Describe other non-monetary sources of support.	
Conflicts of interest	24	Describe any potential for conflict of interest of study contributors in accordance with journal policy. In the absence of a journal policy, we recommend authors comply with International Committee of Medical Journal Editors recommendations.	Page 20, line 429 to 434

For consistency, the CHEERS statement checklist format is based on the format of the CONSORT statement checklist

For peer review only
